# Development and optimization of a differentiated airway epithelial cell model of the bovine respiratory tract

**DOI:** 10.1038/s41598-017-19079-y

**Published:** 2018-01-16

**Authors:** Daniel Cozens, Edward Grahame, Erin Sutherland, Geraldine Taylor, Catherine C. Berry, Robert L. Davies

**Affiliations:** 1 Institute of Infection, Immunity and Inflammation, College of Medical, Veterinary and Life Sciences, University of Glasgow, Glasgow, UK; 2 The Pirbright Institute, Pirbright, Surrey UK; 3 Institute of Molecular, Cell and Systems Biology, College of Medical, Veterinary and Life Sciences, University of Glasgow, Glasgow, UK

## Abstract

Cattle are subject to economically-important respiratory tract infections by various bacterial and viral pathogens and there is an urgent need for the development of more realistic *in vitro* models of the bovine respiratory tract to improve our knowledge of disease pathogenesis. In the present study, we have optimized the culture conditions in serum-free medium that allow bovine bronchial epithelial cells (BBECs) grown at an air-liquid interface to differentiate into a three-dimensional epithelium that is highly representative of the bovine airway. Epidermal growth factor was required to trigger both proliferation and differentiation of BBECs whilst retinoic acid was also essential for mucociliary differentiation. Triiodothyronine was demonstrated not to be important for the differentiation of BBECs. Oxygen concentration had a minimal effect although optimal ciliation was achieved when BBECs were cultured at 14% oxygen tension. Insert pore-density had a significant effect on the growth and differentiation of BBECs; a high-pore-density was required to trigger optimum differentiation. The established BBEC model will have wide-ranging applications for the study of bacterial and viral infections of the bovine respiratory tract; it will contribute to the development of improved vaccines and therapeutics and will reduce the use of cattle in *in vivo* experimentation.

## Introduction

Bovine respiratory disease (BRD) is a multifactorial condition of cattle that involves interactions between different bacterial and viral pathogens and causes significant economic losses to the livestock industries worldwide^1–3^. Commercial vaccines and antibiotics are important tools for the prevention and control of BRD^4–6^. However, vaccines often provide only incomplete or partial protection^7,8^ and the incidence of multi-drug resistant bacterial strains is increasing amid public health concerns associated with the use of antibiotics in food-producing animals^9–11^. Therefore, the development of new or improved vaccines and therapeutics against BRD are urgently required. Currently, progress towards improving our understanding of the pathogenesis of BRD, and developing new and improved vaccines and antimicrobials, is hampered by the lack of physiologically-relevant and reproducible *in vitro* methodologies and an over-emphasis on the use of live animals.

Submerged tissue culture systems, utilizing either immortalized cell lines or primary epithelial cells, are most commonly used for investigating pathogen interactions with the bovine respiratory tract^12–19^. However, the use of submerged cell cultures has numerous limitations: they do not reflect the multicellular complexity of the parental tissue *in vivo*, they lack its three-dimensional architecture, and the physiological conditions are not representative of those found within the respiratory tract. In particular, cells remain undifferentiated in submerged culture and lack many of the characteristics of airway epithelium such as the anatomical barrier function provided by ciliary activity and mucus production^20–23^. To overcome these issues, there has been progress in recent years towards the use of differentiated airway epithelial cells (AECs), grown at an air-liquid interface (ALI), to study the interactions of various bacterial^24–28^ and viral^29–34^ pathogens with the respiratory tracts of different animal species.

The proliferation and differentiation of AECs is a tightly-regulated and complex process requiring the presence of precise concentrations of various growth factors and hormones including insulin, transferrin, hydrocortisone, epinephrine, bovine pituitary extract, triiodothyronine (T3), epidermal growth factor (EGF) and retinoic acid (RA). Of these, EGF plays an important role in cellular proliferation^29,35–37^ whereas RA and T3 are involved in ciliogenesis and mucus production^29,38–41^. It is well recognized that the specific factors, and their concentrations, required for optimal growth and differentiation of AECs vary between species^22,29,37^. Therefore, the optimal culture conditions required for mucociliary differentiation need to be established on a species-by-species basis. Other factors may also influence the proliferation and differentiation of AECs grown at an ALI. These include the composition and pore-density of the insert membranes used for culture^42,43^ and atmospheric oxygen tension during incubation^44–46^.

Establishing an optimal, fully-differentiated *in vitro* airway epithelium is especially important in the context of infection because it is required for adequate development of epithelial barrier function (as reflected in tight junction formation and co-ordinated mucociliary clearance) which is essential as the first line of defence against infection *in vivo*^27,47,48^. Indeed, incomplete or partial differentiation of the airway epithelium can affect the course of infection. Thus, adherence of *Mycoplasma pneumoniae*^27^ and internalization of *Pseudomonas aeruginosa*^49^ are both significantly reduced in differentiated human AECs compared to undifferentiated cells; similarly, influenza A virus replicates within differentiated, but not submerged, swine AECs^29^. The use of differentiated AECs also allows for the identification of specific subsets of cells that are targeted during infection by bacterial^25–27^ or viral^30,34,50–52^ pathogens.

Differentiated AECs have been used to study bacterial^53^ and viral^30,50^ infections of the bovine respiratory tract but, in each of these studies, differentiation was stimulated using the serum replacement Ultroser G and little attention was given to optimizing differentiation of the cells. This is important because serum induces squamous differentiation of AECs and may limit the growth potential and functional characteristics of the cells^54^. Optimal growth and differentiation of AEC cultures are typically achieved through the use of serum-free, hormone-supplemented media^37,55^. Here, we aimed to establish the growth conditions that provide optimal proliferation and differentiation of bovine bronchial epithelial cells (BBECs) grown at an ALI in a defined serum-free medium to produce an AEC model that accurately mimics the *in vivo* bovine respiratory epithelium.

## Results

### Epidermal growth factor influences proliferation and differentiation of BBECs grown at an ALI

**Figure 1:**
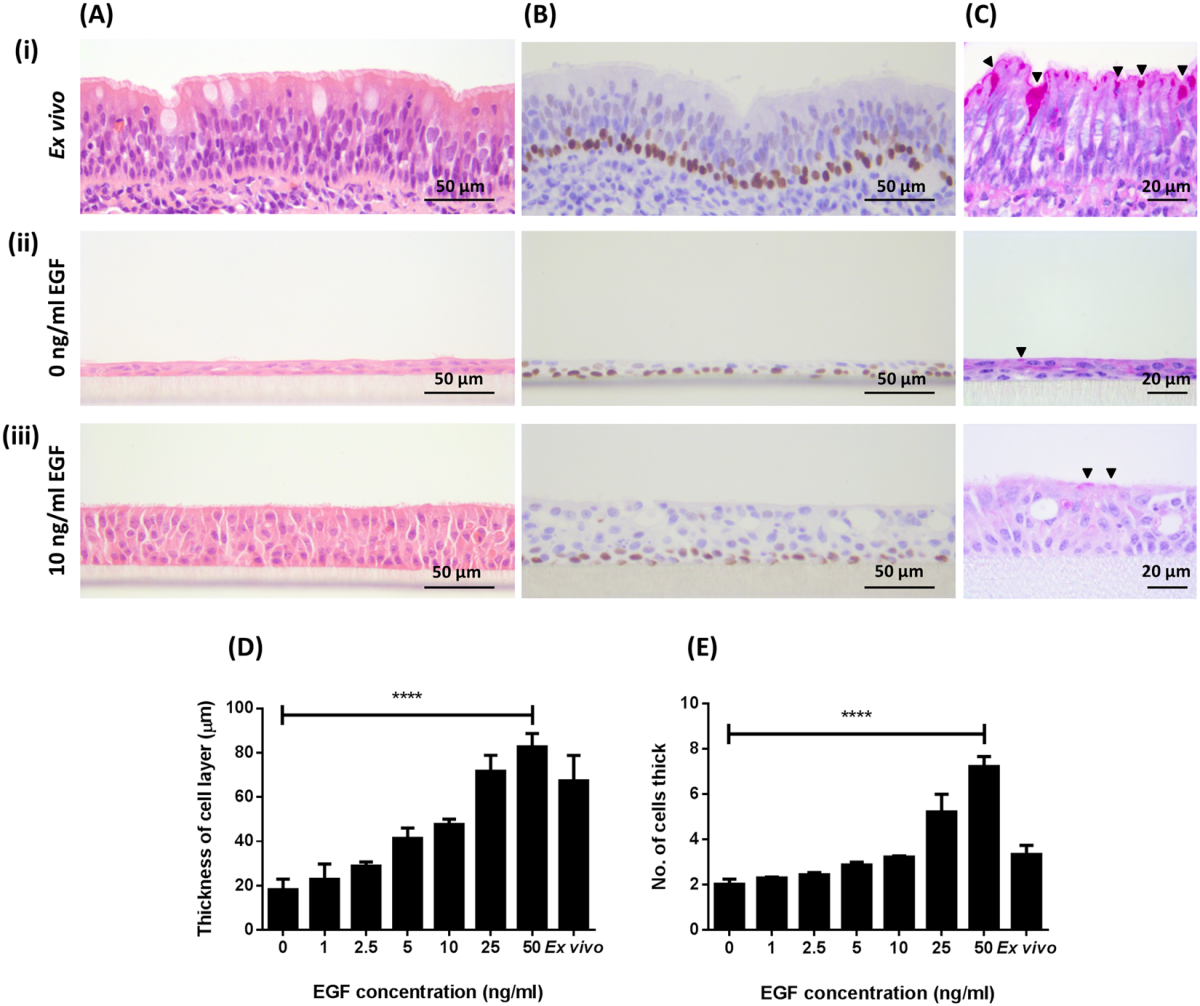
Histological assessment of the effect of EGF on epithelial morphology of BBEC cultures. BBEC cultures were grown for 21 days at an ALI with varying concentrations of EGF before being fixed and paraffin-embedded using standard histological techniques; samples of *ex vivo* tissue were also taken from the donor animal. Sections were cut, deparaffinised and stained using (**A**) H&E, (**B**) immunohistochemical-labelling of basal cells (p63-labelled cells display brown nuclei) and (**C**) PAS (black arrowheads indicate goblet cells). Representative images are shown of (i) *ex vivo* bovine bronchial epithelium, and BBECs grown in the presence of (ii) 0 and (iii) 10 ng/ml EGF (see Fig. S1). Quantitative analysis (using ImageJ) of histological sections of BBEC layers grown in the presence of 0, 1.0, 2.5, 5.0, 10.0, 25.0 and 50.0 ng/ml EGF (see Fig. S1A), and *ex vivo* tissue, showing (**D**) epithelial thickness and (**E**) the number of cell layers comprising the epithelium was performed. For each insert, three measurements were taken (left, centre and right) in each of five 400x fields of view evenly distributed across the sample; three inserts were analysed per growth condition and the data represents the mean +/− standard deviation from tissue derived from three different animals. Statistical significance was tested using an Ordinary one-way ANOVA: **** = P < 0.0001.

Bovine bronchial epithelial cells were grown at an ALI for 21 days in medium containing 100 nM RA and with concentrations of EGF ranging from 0 to 50 ng/ml. Proliferation of BBECs was dependent on the presence and concentration of EGF as assessed by epithelial thickness and morphology (Figs. 1A and S1A). In the absence of EGF, BBECs grew as thin, squamous layers with large proportions of the cultures forming monolayers (Fig. 1A [ii]). However, supplementation with EGF induced the development of a pseudostratified, columnar morphology (Fig. 1A [iii]) that was reminiscent of the *ex vivo* tissue (Fig. 1A [i]). Epithelial thickness (Fig. 1D) and the number of cells within the epithelium (Fig. 1E) increased with increasing EGF concentration (Fig. S1A). Thus, there was a direct correlation between EGF concentration and cellular proliferation within the epithelial layer (p < 0.0001, Ordinary one-way ANOVA). The overall morphology of the epithelium was also dependent on EGF concentration (Fig. S1A). Cells were cuboidal when grown in the presence of 1.0 and 2.5 ng/ml EGF (Figs. S1A [ii] and [iii]) but had a more columnar morphology in the presence of 5.0 and 10.0 ng/ml EGF (Figs. S1A [iv] and [v]) which more closely replicated the *ex vivo* tissue. Conversely, in cultures maintained at 25 and 50 ng/ml EGF (Figs. S1A [vi] and [vii]), the epithelial morphology was increasingly less uniform, having a more irregular architecture as opposed to the stereotypical pseudostratified epithelium observed in *ex vivo* tissue (Fig. 1A [i]). The increased irregularity at 25 and 50 ng/ml EGF was accompanied by a corresponding increase in signs of cellular and tissue deterioration. In particular, there was a positive correlation between EGF concentration and the numbers of pyknotic nuclei and vacuoles observed within the tissue (Fig. S2; p < 0.001, Ordinary one-way ANOVA). The transcription factor p63 was used as a marker to identify basal cells; p63 is highly-expressed in the basal cells of epithelial tissues and is commonly used as a specific marker of this progenitor cell type^48,56–58^. Basal cells constituted a well-defined, single continuous layer attached to the basement membrane within the *ex vivo* tissue (Fig. 1B [i]). Similarly, basal cells comprised a single row at the interface between the epithelial layer and insert membrane in the BBEC cultures grown in both the absence (Fig. 1B [ii]) and presence (Fig. 1B [iii]) of EGF. The distribution of basal cells remained consistent at all EGF concentrations although small numbers of basal cells were observed within the suprabasal layer at EGF concentrations of 25 and 50 ng/ml (Figs. S1B [vi] and [vii]).

**Figure 2:**
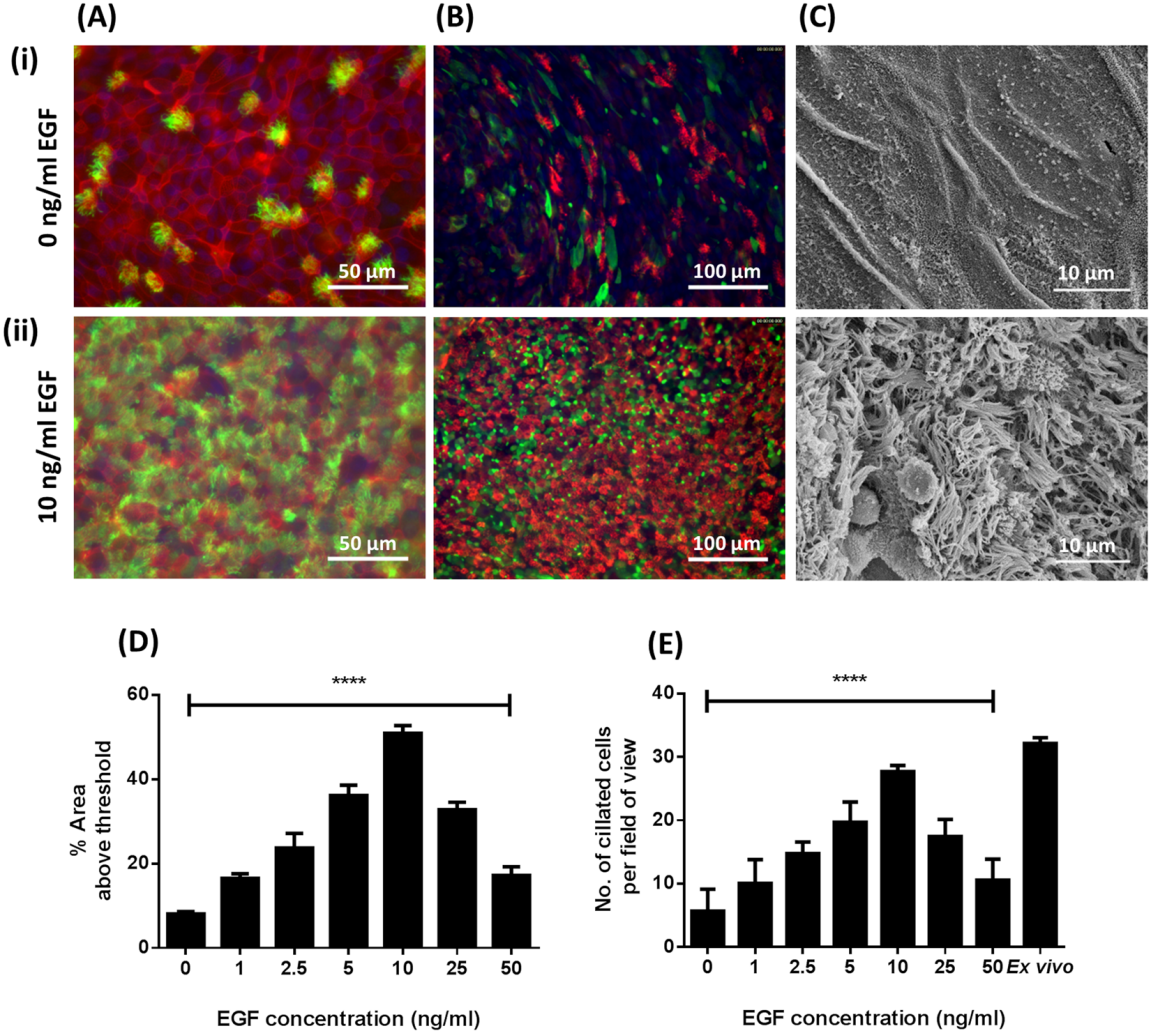
Effect of EGF on cell differentiation of BBEC cultures. BBEC cultures were grown for 21 days at an ALI with varying concentrations of EGF before fixation. The BBEC cultures were subsequently immunostained to assess (**A**) ciliation (cilia - green; F-actin - red; nuclei - blue) and (**B**) mucus production (mucus - green; cilia - red; nuclei - blue) or (**C**) examined by SEM. Representative images are shown of BBECs grown in the presence of (i) 0 and (ii) 10 ng/ml EGF (see Figs. S3A, B and D). Quantitative analysis of ciliation of the apical surface of BBEC cultures grown in the presence of 0, 1.0, 2.5, 5.0, 10.0, 25.0 and 50.0 ng/ml EGF was performed using (**D**) fluorescence intensity thresholding of immunostained cultures (see Fig. S3A) and (**E**) by counting the number of ciliated cells per field of view in H&E-stained sections (see Fig. S1A). In (**D**), ciliation was quantified by measuring the area above a fluorescence intensity threshold in ImageJ; for each insert, five regions evenly distributed across the sample were measured. In (**E**), for each insert, ciliated cells were counted in each of five 400x fields of view evenly distributed across the sample. For all of the above quantifications, three inserts were analysed per growth condition and the data represents the mean +/− standard deviation from tissue derived from three different animals. Statistical significance was tested using an Ordinary one-way ANOVA: **** = P < 0.0001.

Ciliation was also dependent on the presence of EGF. Histological assessment (Fig. 1A [ii]), immunofluorescence-microscopy (Fig. 2A [i]), and scanning electron microscopy (SEM) (Fig. 2C [i]) of BBEC cultures identified no, or very few, ciliated cells in cultures grown in the absence of EGF. Conversely, the same approaches identified abundant cilia in the presence of EGF (Figs. 1A [iii], 2A [ii] and C [ii]). Quantitation of ciliation in histological samples (Fig. S1A) and immunostained cultures (Fig. S3A) demonstrated that ciliation increased with increasing concentrations of EGF and peaked at 10 ng/ml, declining thereafter (Figs. 2D and E). These observations were confirmed by SEM (Fig. S3D). Indeed, most of the apical surface was composed of ciliated cells (Figs. S3A [v] and D [v]) at an EGF concentration of 10 ng/ml and this observation mimicked the morphology of the *ex vivo* epithelium.

A similar correlation was observed in the relationship between EGF concentration and the differentiation of mucus-producing goblet cells. These cells were identified in *ex vivo* tissue (Fig. 1C [i]) and also in BBECs grown in the presence of EGF using Periodic acid-Schiff (PAS)-staining of histological sections (Fig. 1C [iii]) and Jacalin-labelling. Cultures maintained in the absence of EGF produced few cells positive for mucus production (Fig. 2B [i]) whereas the presence of EGF resulted in abundant staining of mucus-producing cells (Fig. 2B [ii]). Although goblet cells were identified in the presence of all concentrations of EGF by PAS-staining, it was difficult to make any conclusions about concentration effects (Fig. S1C). However, Jacalin-labelling was more effective and indicated an increase in Muc5Ac-positive cells as EGF concentration increased, peaking at a concentration of 10 ng/ml (Fig. S3B).

**Figure 3:**
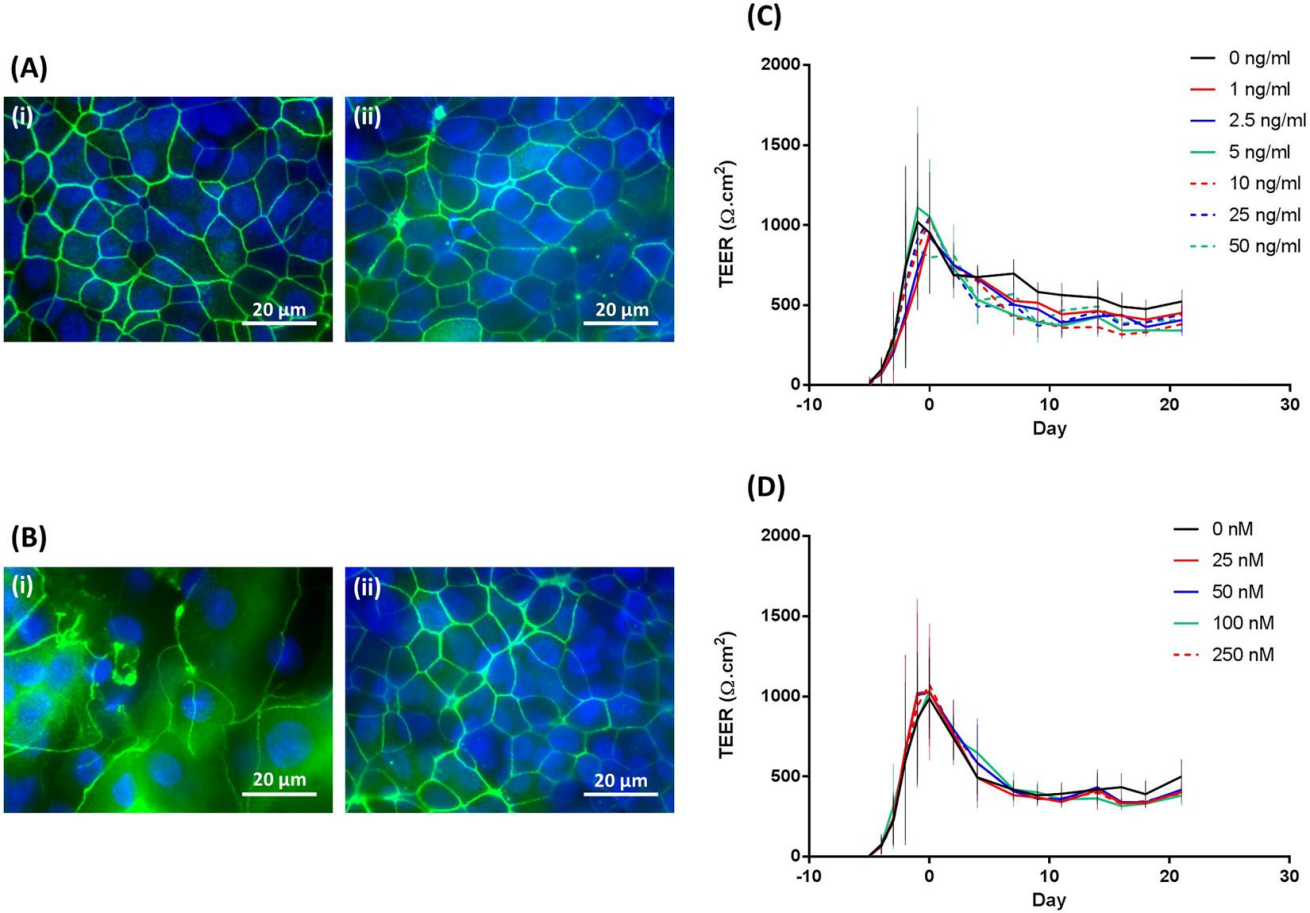
Effect of EGF and RA on the barrier properties of BBEC cultures. BBEC cultures were grown for 21 days at an ALI with varying concentrations of EGF or RA before fixation. Tight-junction formation of the BBEC cultures grown in the presence of (**A**) EGF or (**B**) RA was subsequently assessed by immunostaining (tight-junctions - green; nuclei - blue). Representative images are shown of BBECs grown in the presence of (i) 0 and (ii) 10 ng/ml EGF in (**A**) (see Fig. S3C) and (i) 0 and (ii) 100 nM RA in (**B**) (see Fig. S5C). Tight-junction integrity during the course of epithelial cell proliferation and differentiation was also assessed by measuring the TEER of BBEC cultures grown in the presence of (**C**) 0, 1.0, 2.5, 5.0, 10.0, 25.0 and 50.0 ng/ml EGF and (**D**) 0, 25, 50, 100 and 250 nM RA. Three inserts were analysed per growth condition and the data represents the mean +/− standard deviation from tissue derived from three different animals.

The EGF concentration had limited effect on tight junction formation. Tight junctions were formed, with no differences in ZO-1 staining, in both the absence (Fig. 3A [i]) and presence (Fig. 3A [ii]) of EGF. Indeed, there were no differences in ZO-1 staining at any of the EGF concentrations used (Fig. S3C). In support of these data, there were no significant differences in trans-epithelial electrical resistance (TEER) measurements for different EGF concentrations over the 21-day course of culture growth (Fig. 3C). In all cases, TEER increased rapidly during the submerged stage of culture and gradually declined thereafter during the ALI phase.

### Retinoic acid influences differentiation of BBECs grown at an ALI

**Figure 4:**
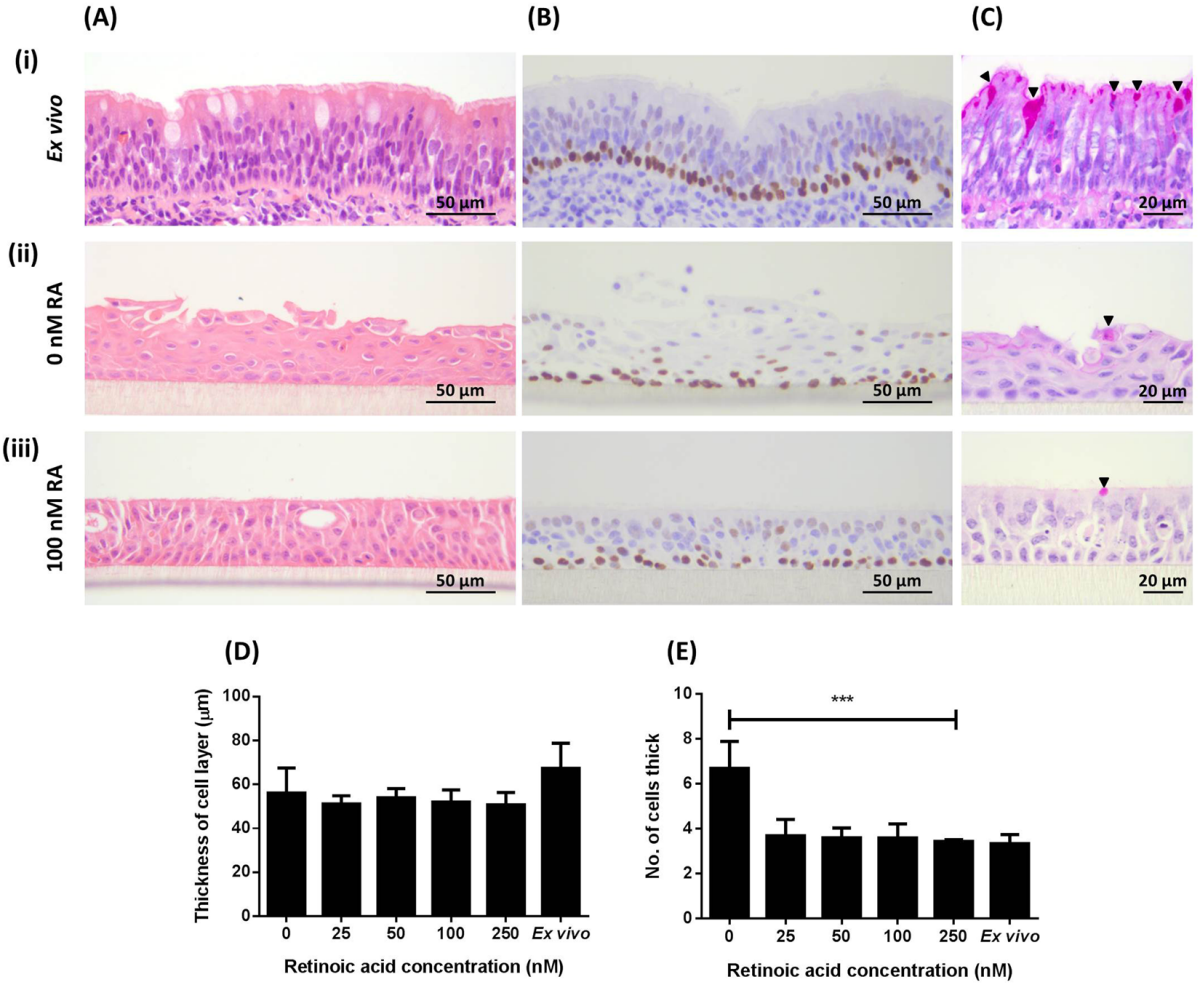
Histological assessment of the effect of RA on epithelial morphology of BBEC cultures. BBEC cultures were grown for 21 days at an ALI with varying concentrations of RA before being fixed and paraffin-embedded using standard histological techniques; samples of *ex vivo* tissue were also taken from the donor animal. Sections were cut, deparaffinised and stained as described in Fig. 1. Representative images are shown of (i) *ex vivo* bovine bronchial epithelium, and BBECs grown in the presence of (ii) 0 and (iii) 100 nM RA (see Fig. S4). Quantitative analysis (using ImageJ) of histological sections of BBEC layers grown in the presence of 0, 25, 50, 100 and 250 nM RA (see Fig. S4A), and *ex vivo* tissue, to assess (**D**) epithelial thickness and (**E**) the number of cell layers comprising the epithelium, was performed as described in Fig. 1. Statistical significance was tested using an Ordinary one-way ANOVA: *** = P < 0.001.

Bovine bronchial epithelial cells were grown at an ALI for 21 days in medium containing 10 ng/ml EGF and with concentrations of RA ranging from 0 to 250 nM. The BBEC cultures formed a thick, stratified epithelium of squamous cells in the absence of RA (Fig. 4A [ii]) whereas a pseudostratified layer was formed in the presence of RA (Fig. 4A [iii]) that was similar to the *ex vivo* epithelium (Fig. 4A [i]). However, histological comparison of the cultures grown in the presence of increasing RA concentrations (Fig. S4A) indicated little variation in either the thickness of the epithelial layer (Fig. 4D), the number of cells within the layer (Fig. 4E), the cell size and shape, and overall tissue architecture (Fig. S4A), or the numbers of pyknotic nuclei and vacuoles present (results not shown). Basal cells comprised a single row at the interface between the epithelium and insert membrane in BBEC cultures grown in both the absence (Fig. S4B [ii]) and presence (Fig. 4B [iii]) of RA, mimicking that observed in the *ex vivo* tissue (Fig. 4B [i]), and their distribution within the BBEC cultures remained consistent at all concentrations of RA (Fig. S4B).

**Figure 5:**
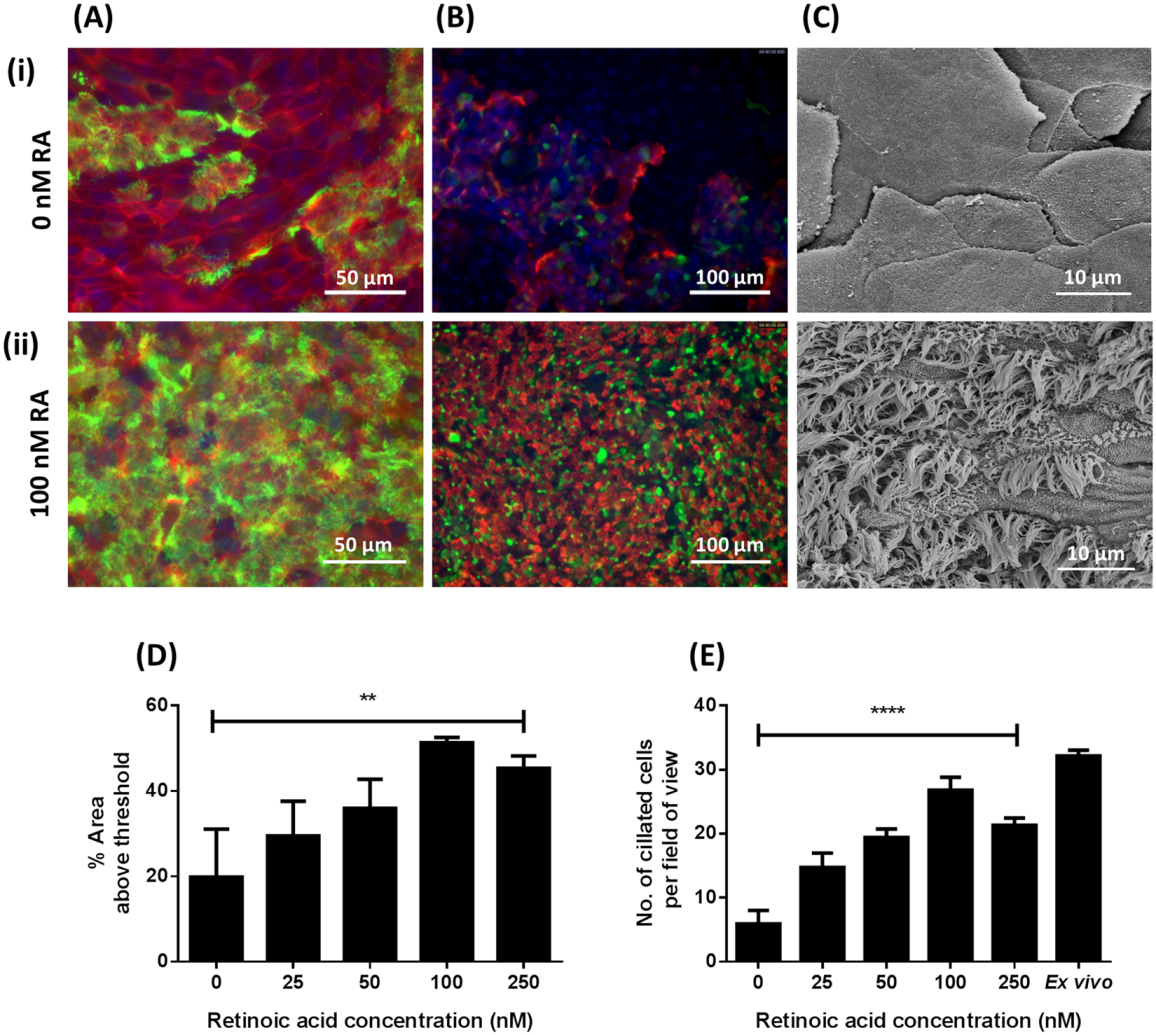
Effect of RA on cell differentiation of BBEC cultures. BBEC cultures were grown for 21 days at an ALI with varying concentrations of RA before fixation. The BBEC cultures were subsequently immunostained to assess (**A**) ciliation (cilia - green; F-actin - red; nuclei - blue) and (**B**) mucus production (mucus - green; cilia - red; nuclei - blue) or (**C**) examined by SEM. Representative images are shown of BBECs grown in the presence of (i) 0 and (ii) 100 nM RA (see Figs. S5A, B and D). Quantitative analysis of ciliation of the apical surface of BBEC cultures grown in the presence of 0, 25, 50, 100 and 250 nM RA was performed using (**D**) fluorescence intensity thresholding of immunostained cultures (see Fig. S5A) and (**E**) by counting the number of ciliated cells per field of view in H&E-stained sections (see Fig. S4A) as described in Fig. 2. Statistical significance was tested using an Ordinary one-way ANOVA: ** = P < 0.01; **** = P < 0.0001.

Histological assessment (Fig. 4A [ii]) and immunostaining (Fig. 5A [i]) of BBEC cultures identified no or very few ciliated cells when grown in the absence of RA; SEM confirmed these observations which highlighted the undifferentiated, squamous, non-ciliated nature of the epithelial cells when grown in the absence of RA (Fig. 5C [i]). Conversely, the same approaches identified the presence of cilia in BBECs grown in media supplemented with RA (Figs. 4A [iii], 5A [ii] and C[ii]). Quantitation of ciliation in histological samples (Fig. S4A) and immunostained cultures (Fig. S5A) demonstrated that ciliation increased with increasing concentrations of RA and peaked at 100 nM, declining thereafter (Figs. 5D and E). There was a significant positive correlation between RA concentration and ciliation in immunostained cultures (Fig. 5D; p < 0.01, Ordinary one-way ANOVA) and in haematoxylin and eosin (H&E)-stained sections (Fig. 5E; p < 0.0001, Ordinary one-way ANOVA). These observations were confirmed by SEM (Fig. S5D). Indeed, most of the apical surface was composed of ciliated cells (Figs. S5A[iv] and D[iv]) at a RA concentration of 100 nM, which was reminiscent of *ex vivo* tissue.

The differentiation of goblet cells and production of mucus were similarly dependent on RA concentration. Mucus-producing goblet cells were identified in BBECs grown in the presence of RA using PAS-staining (Fig. 4C), although it was difficult to make any conclusions about concentration effects (Fig. S4C). Cultures maintained in the absence of RA produced few cells positive for mucus production by Jacalin-labelling (Fig. 5B [i]) whereas the presence of RA resulted in staining of abundant mucus-producing cells (Fig. 5B [ii]); indeed, Jacalin-labelling demonstrated an increase in Muc5Ac-positive cells as RA concentration increased, peaking at a concentration of 100 nM (Fig. S5B).

The presence of RA had little effect on tight junction formation. Tight junctions were formed, with no differences in ZO-1 staining, in both the absence (Fig. 3B [i]) and presence (Fig. 3B [ii]) of RA, and at all RA concentrations used (Fig. S5C). In support of these data, there were no significant differences in TEER measurements for different RA concentrations over the 21-day course of cell culture (Fig. 3D). In all cases, TEER rapidly increased during the submerged stage of culture and gradually declined thereafter during the ALI phase.

### Triiodothyronine has no effect on BBECs grown at an ALI

Bovine bronchial epithelial cells were grown at an ALI for 21 days in media supplemented with 10 ng/ml EGF, 100 nM RA, and in the absence or presence (6.7 ng/ml) of T3. The addition of T3 had no effect on the overall morphology (Fig. S6A) or thickness of the epithelial layers (Fig. S6D), the number of cells in the layers (Fig. S6E), or the distribution of basal cells (Fig. S6B). The degree of ciliation as determined by histology (Fig. S6A) and immunofluorescence microscopy (Fig. S7A) was not affected by the presence of T3 (Figs. S7E and F), and this was confirmed by SEM (Fig. S7D). Similarly, the presence of mucus-producing goblet cells, as assessed by PAS-staining (Fig. S6C) and Jacalin-labelling (Fig. S7B), was not affected by T3, and these observations were confirmed by SEM (Fig. S7D). Finally, T3 had no effect on tight junction formation as evidenced by ZO-1 staining (Fig. S7C).

### High pore density cell culture inserts are required for optimum epithelial morphology and differentiation of BBECs grown at an ALI

**Figure 6:**
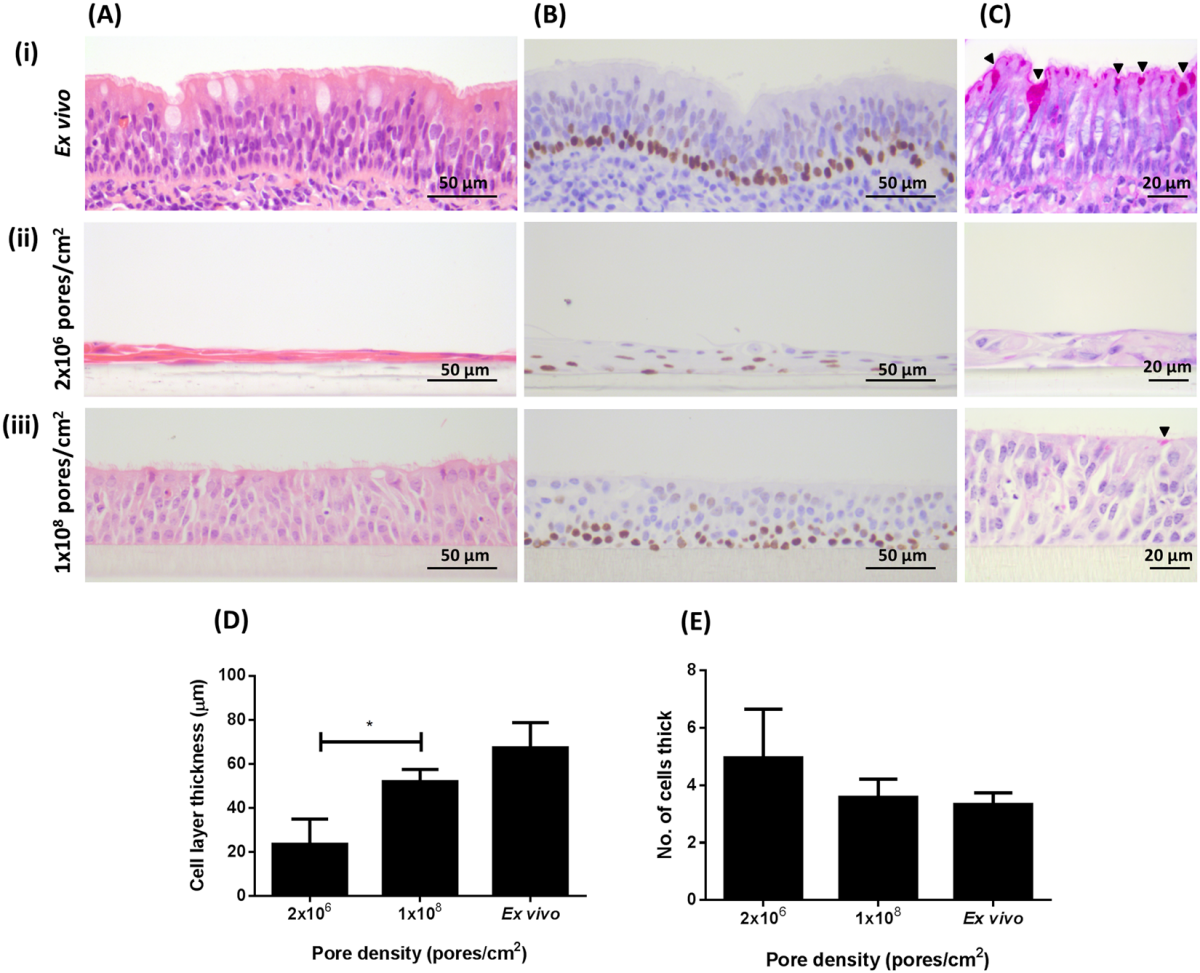
Histological assessment of the effect of cell culture insert pore density on epithelial morphology of BBEC cultures. BBEC cultures were grown for 21 days at an ALI on membranes with pore densities of 2.0 × 10^6^ or 1.0 × 10^8^ pores/cm^2^ before being fixed and paraffin-embedded using standard histological techniques; samples of *ex vivo* tissue were also taken from the donor animal. Sections were cut, deparaffinised and stained as described in Fig. 1. Representative images are shown of (i) *ex vivo* bovine bronchial epithelium, and BBECs grown on inserts with (ii) 2.0 × 10^6^ and (iii) 1.0 × 10^8^ pores/cm^2^. Quantitative analyses (using ImageJ) of histological sections of BBEC layers, and *ex vivo* tissue, to assess (**D**) epithelial thickness and (**E**) the number of cell layers comprising the epithelium, were performed as described in Fig. 1. Statistical significance was tested using an Ordinary one-way ANOVA: * = P < 0.05.

**Figure 7:**
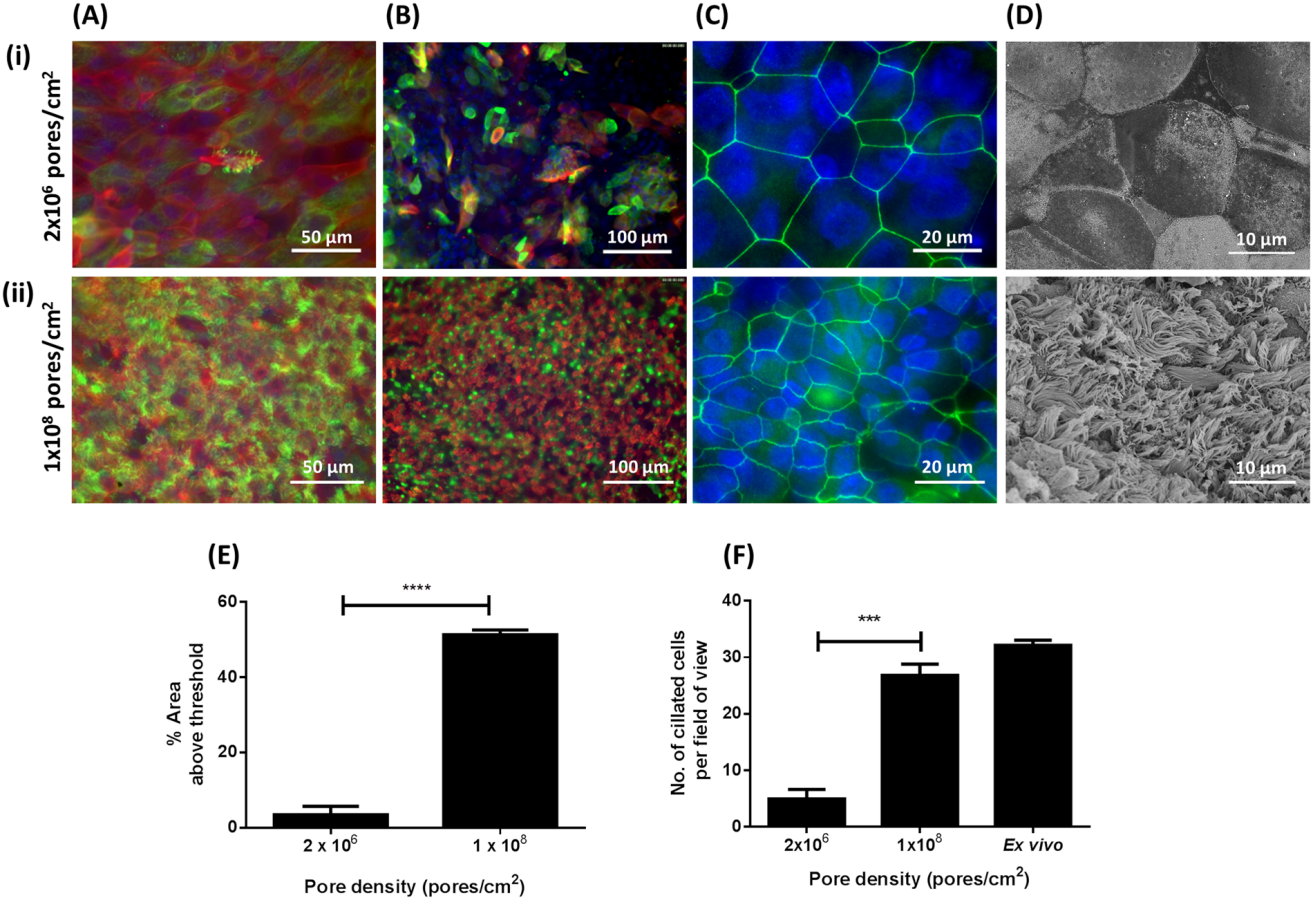
Effect of cell culture insert pore density on cell differentiation. BBEC cultures were grown for 21 days at an ALI on membranes with pore densities of 2.0 × 10^6^ or 1.0 × 10^8^ pores/cm^2^ before fixation. The BBEC cultures were subsequently immunostained to assess (**A**) ciliation (cilia - green; F-actin - red; nuclei - blue), (**B**) mucus production (mucus - green; cilia - red; nuclei - blue) and (**C**) tight-junction formation (tight-junctions - green; nuclei - blue) or (**D**) examined by SEM. Representative images are shown of BBECs grown on inserts with (i) 2.0 × 10^6^ and (ii) 1.0 × 10^8^ pores/cm^2^. Quantitative analysis of ciliation of the apical surface of BBEC cultures was performed using (**E**) fluorescence intensity thresholding of immunostained cultures and (**F**) by counting the number of ciliated cells per field of view in H&E-stained sections as described in Fig. 2. Statistical significance was tested using an Ordinary one-way ANOVA: *** = P < 0.001; **** = P < 0.0001.

Bovine bronchial epithelial cells were cultured at an ALI for 21 days in culture medium containing 10 ng/ml EGF and 100 nM RA on polyethylene terephthalate (PET) membranes having pore densities of either 2.0 × 10^6^ pores/cm^2^ (low-pore-density [LPD]) or 1.0 × 10^8^ pores/cm^2^ (high-pore-density [HPD]). Cells grown on LPD inserts formed a stratified, squamous epithelium (Fig. 6A [ii]) and failed to replicate the *ex vivo* epithelial morphology (Fig. 6A [i]), whereas cells cultured on the HPD inserts formed a columnar, pseudostratified epithelium (Fig. 6A [iii]) that was more reminiscent of the *ex vivo* epithelium. The epithelium grown on the HPD inserts was of significantly greater thickness (Fig. 6D) but comprised a smaller number of cells (Fig. 6E) than that grown on the LPD inserts. The stratified epithelium characteristic of the LPD inserts possessed several layers of p63^+^ basal cells (Fig. 6B [ii]) in contrast to the pseudostratified-type epithelium generated on the HPD membranes which possessed a well-defined single layer of basal cells (Fig. 6B [iii]) characteristic of the *ex vivo* epithelium (Fig. 6B [i]). The degree of ciliation on LPD and HPD inserts was assessed by histology (Fig. 6A) and immunofluorescence microscopy (Fig. 7A). In both cases, the number of ciliated cells on the LPD inserts was significantly lower than the number on the HPD inserts (Figs. 7E and F). These observations were confirmed by SEM (Fig. 7D); cells grown on LPD inserts were large, squamous and non-ciliated (Fig. 7D [i]) whereas cells grown on HPD inserts were well-differentiated and highly ciliated (Fig. 7D [ii]). Jacalin-labelling indicated little evidence of mucus production by epithelial cells grown on the LPD inserts (Fig. 7B [i]) whereas there was significant mucus production by cells grown on the HPD inserts (Fig. 7B [ii]). Tight junction formation was unaffected by membrane pore density based on ZO-1 staining, although the difference in cell size between squamous cells grown on LPD inserts (Fig. 7C [i]) and well-differentiated cells grown on HPD inserts (Fig. 7C [ii]) was very apparent.

### Oxygen tension affects differentiation of BBECs grown at an ALI

Bovine bronchial epithelial cells were cultured at an ALI for 21 days in medium containing 10 ng/ml EGF and 100 nM RA in the presence of 7, 14 or 21% oxygen tension. Epithelia grown at these different oxygen tensions were of very similar histological appearance having a well differentiated pseudostratified architecture in each case (Fig. S8A). The epithelial layers were of similar thickness although the cultures grown in the presence of 14% oxygen were of marginally higher thickness (Figs. S8D and E). The location and appearance of the basal cell layers were very similar in each case (Fig. S8B). The degree of ciliation was assessed by histology (Fig. S8A) and immunofluorescence microscopy (Fig. S9A). Cultures exposed to 14 and 21% oxygen exhibited a statistically significant higher percentage of ciliated cells compared to cultures exposed to 7% oxygen as determined by immunostaining (Fig. S9E) although this effect was relatively minor; highest ciliation occurred in cultures grown in the presence of 14% oxygen. High levels of ciliation were observed by SEM at all oxygen tensions (Fig. S9D). Oxygen tension had no demonstrable effect on other markers of differentiation including mucus production, as assessed by PAS-staining (Fig. S8C) and Jacalin-labelling (Fig. S9B), or tight-junction formation (Fig. S9C).

## Discussion

The aim of the present study was to develop and optimize a differentiated AEC model that mimics the bovine respiratory epithelium *in vivo*. To achieve this, optimum concentrations of key growth factors known to be involved in the proliferation and differentiation of AECs, including EGF and RA^35,37–40,59–62^, were determined in a defined, serum-free medium; we also examined the effects of T3, insert membrane pore density and atmospheric oxygen tension. Differentiated BBEC cultures were characterized using established markers of differentiation and a set of growth conditions identified that produced an AEC model most closely representative of the bovine respiratory epithelium.

Epidermal growth factor influences cell proliferation and differentiation by activating the EGF receptor (EGFR)^63,64^. Activation of EGFR triggers epithelial cell proliferation and mucin synthesis within the respiratory epithelium^65,66^. These phenotypic effects have previously been replicated *in vitro*; EGF influences both epithelial thickness and mucin production of AEC cultures grown at an ALI^29,35,36,39,59^. However, the effect of EGF on AECs varies considerably among different animal species. For example, removal of EGF from the culture medium increased mucin synthesis and decreased cell proliferation of human AECs^39^, decreased mucin production and increased ciliation of rat tracheal epithelial cells^36,59^ and increased both cell proliferation and ciliation in swine AECs^29^. The optimum concentration of EGF used for the culture of AECs also varies considerably between species^29,35,36,59^.

In the present study, EGF was similarly shown to have a major influence on the proliferation and differentiation of BBECs. Epithelial cell proliferation was directly proportional to the concentration of EGF to which the cells were exposed. However, increased epithelial cell proliferation at higher concentrations of EGF was not due to basal cell hyperplasia^67^, as previously described in human AECs^35^, because the number of p63^+^ cells was consistent at all concentrations of EGF. Rather, increased epithelial thickness and cell numbers were due to an increased rate of proliferation occurring from the basal cells. Higher concentrations of EGF (e.g. 25 to 50 ng/ml) were associated with increased disorder of epithelial morphology and with increased numbers of aberrantly-located p63^+^ cells, pyknotic cells and vacuoles within the epithelium. These findings suggest an increase in cell death due to apoptosis and/or autophagy at higher EGF concentrations^68,69^. Taken together, these results clearly indicate that overexposure to EGF has a detrimental effect on the overall health of the epithelium. Similar untoward effects due to high EGF concentrations have been described in AECs in other animal species^35,37^. In addition to proliferation, EGF was also clearly involved in differentiation of BBECs because increased concentrations of EGF resulted in increased numbers of fully differentiated ciliated and goblet cells at the apical surface. However, there was little observable effect of EGF on tight junctions suggesting that these are formed at a very early stage of epithelial cell proliferation and are independent of differentiation^27^, as indicated by their presence in submerged, undifferentiated cultures^70,71^. Overall, our data indicated that ciliation of the apical surface and mucus production peaked at an EGF concentration of 10 ng/ml.

It is well documented that retinoids play important roles in the maintenance of mucociliary differentiation of tracheobronchial epithelium in various animal species^35,38,60–62^. Animals deficient in vitamin A, a precursor of RA, display squamous metaplasia of various epithelia, including those of the respiratory and urinary tracts; such epithelia have a stratified, squamous morphology^72,73^. Similarly, airway epithelia cultured *in vitro* in serum-free media in the absence of RA possess a stratified, squamous morphology^29,35,61,62^. Conversely, a stereotypical differentiated columnar epithelium is formed, accompanied by an increase in the number of ciliated cells and an increase in mucus production, in AEC cultures in the presence of RA^29,35,38,61^.

In the present study, RA had similar effects on BBEC cultures. Thus, BBECs formed a squamous, stratified epithelium that resembled the squamous metaplasia phenotype^74^ in the absence of RA, whereas they formed a stereotypical pseudostratified, columnar epithelium, similar to that exhibited in the *ex vivo* tissue, in the presence of RA. In contrast to EGF, there was no variation in epithelial thickness and morphology at different RA concentrations. However, ciliation was more extensively affected by RA concentration, increasing as RA concentration increased and peaking at a concentration of 100 nM; this trend was confirmed by histological analysis, immunofluorescence microscopy and SEM. These findings clearly indicate that RA plays an important role in controlling differentiation of BBECs to a ciliated phenotype and are in agreement with previous observations^29,35,38,60^. Retinoic acid also stimulated differentiation of BBECs to a mucus-producing phenotype. The numbers of mucus-producing goblet cells were not as abundant in the differentiated BBEC cultures as they were in the *ex vivo* tissue but a similar trend has previously been demonstrated by others in bovine tissue^53^. As was the case for EGF, RA had very little effect on tight junction formation. Overall, these observations agree with those findings described above pertaining to the role of RA in stimulating ciliation and mucus production in AECs from different species. Notably, a RA concentration of 100 nM resulted in an epithelial layer that most closely resembled that of the *ex vivo* tissue.

The thyroid hormone T3 is involved in the regulation of mucin synthesis in epithelial cells grown at an ALI^35,38,39^. The absence of T3 induces mucus secretion in human bronchial epithelial cells^38,39^ which may be due to the downregulation of RA receptors^35,40^, suggesting a complex signalling pathway involving both RA and T3. Removal of T3 from the growth medium resulted in an increase in MUC5AC but not MUC2 mRNA expression in human AECs^38^. The presence of Muc5Ac on AECs grown at an ALI has previously been demonstrated by labelling with Jacalin, a lectin which binds specifically to O-glycoproteins^75,76^. In the present study, PAS-staining, Jacalin-labelling and SEM analysis all demonstrated that removal of T3 from the culture medium had no noticeable effect on mucus production; there were also no observable differences in epithelial morphology or degree of ciliation. Overall, these results demonstrated that the presence of T3 in the culture medium does not down-regulate mucus production in BBECs as it does in AECs from other species^38,39^. These findings provide further evidence that the regulatory mechanisms involved in the differentiation of respiratory epithelium exhibit species-to-species variation^22,29,37^.

The physical nature of the porous membranes used to grow differentiated AECs at an ALI has received little attention although membranes manufactured from polycarbonate^53,77^, polytetrafluoroethylene (PTFE)^36,78^ and PET^27,35,38,50,76^ have been used in various studies. Different pore-size and pore-densities are also available and membranes may be coated with collagen or other extra-cellular matrix components. A pore-size of 0.4 µm is used almost exclusively for the culture of AECs and membranes are often, but not always, collagen-coated. However, the pore-density of inserts used to culture AECs is rarely, if ever, reported in the literature. Widdicome *et al*.^42^ demonstrated that growth and differentiation of human tracheal epithelial cells was far superior on porous compared to solid supports. These authors concluded that the squamous morphology of cells grown on solid supports was likely a combination of immersion feeding as well as depletion of basolateral nutrients. Since porous membranes allow multidirectional exposure to nutrients and waste products it is likely that pore density itself is important. This clearly becomes more of an issue for cells growing at an ALI because they can only obtain nutrients from the basolateral medium. To our knowledge, the effect of pore density on the culture of AECs grown at an ALI has not previously been investigated although Lee *et al*.^43^ demonstrated that optimal growth of human embryonic stem cells occurred on PET membranes with a pore density of 1–4 × 10^6^ pores/cm^2^.

In the present study, we demonstrated that pore density had a significant effect on the morphology and differentiation of BBECs grown at an ALI. On LPD membranes, the cultures grew as a squamous, non-ciliated, stratified epithelium; they also contained an increased number of layers of p63^+^ cells suggestive of basal cell hyperplasia^67^. In contrast, HPD membranes resulted in a well-developed pseudostratified morphology and excellent mucociliary differentiation. These data clearly demonstrate that a HPD membrane is necessary for optimal differentiation of BBECs grown at an ALI. As previously suggested with regard to solid surfaces^42^, it is likely that LPD membranes do not allow adequate transport of nutrients to support normal growth and differentiation of epithelial cells on the apical surface. The absence of medium on the apical surface and complete dependence on nutrients within the basolateral medium suggests that adequate transport across the membrane is of paramount importance to the health of cells growing in an ALI environment and is an important consideration in ALI cultures.

Oxygen tension plays an important role in controlling cell proliferation, fate and morphogenesis during the development of many tissues^44^. Bone marrow mesenchymal stem cells and mouse foetal cortical neural stem cells cultured at 5% O_2_ out-performed cells grown at 20 or 21% O_2_^45,46^. Oxygen tension within the respiratory tract ranges from 14.5% in the alveoli to 19.7% in inspired air within the trachea^79^. We hypothesized that AEC proliferation and differentiation may be affected by variation in oxygen tension. Bovine bronchial epithelial cells exhibited little variation in morphology or differentiation at different oxygen tensions although cultures grown in 14% oxygen were marginally thicker and had slightly increased ciliation compared to those grown in 7 and 21% oxygen. Further analyses of selected molecular markers might reveal more subtle variation^45,46^ but, for these reasons, BBECs were routinely cultured in an atmosphere of 14% oxygen tension.

In conclusion, we have optimized culture conditions for the successful establishment of a differentiated AEC model of the bovine respiratory tract in a defined, serum-free medium. Optimization was performed using a range of recognized markers of cell proliferation and differentiation such that the model closely replicates airway epithelium *in vivo*. Overall, epithelial architecture and morphology was a remarkable mimic of that of *ex vivo* tissue when the cultures were grown on HPD inserts at optimal EGF and RA concentrations of 10 ng/ml and 100 nM, respectively. The optimization of this bovine AEC model will provide a tool that can be widely used for studying host-pathogen interactions involved in BRD and, ultimately, in the development of new and improved vaccines and therapeutics. Importantly, the future deployment of this three-dimensional model of the bovine respiratory tract is likely to reduce the use of *in vivo* studies involving cattle and will impact on the 3Rs.

## Methods

### Isolation of bovine bronchial epithelial cells

Bronchial epithelial cells were isolated from the lungs of freshly-slaughtered cattle aged 18- to 36-months (obtained from Sandyford Abattoir Ltd., Paisley, UK). The lungs were transported to the laboratory on ice, the left and right bronchi dissected from below the bifurcation of the trachea, and surrounding tissue removed. Small (~1 cm^2^) sections of bronchial tissue were fixed in 2% (w/v) formaldehyde for histological analysis and the bronchi swabbed for bacterial/fungal contamination. The bronchi were cut into three sections (~6–7 cm in length) and each of these was cut twice longitudinally to yield two rectangular tissue pieces. The bronchial sections were incubated overnight at 4 °C in “digestion medium” (DM) which comprised a 50:50 mixture of Dulbecco’s modified Eagle’s medium (DMEM) and Ham’s nutrient F-12 containing 1 mg/ml dithioreitol, 10 µg/ml DNAase and 1 mg/ml Protease XIV (from *Streptomyces griseus*) and supplemented with penicillin (100 U/ml), streptomycin (100 µg/ml) and amphotericin (2.5 µg/ml). All subsequent media were also supplemented with penicillin-streptomycin and amphotericin. The protease was neutralized by the addition of foetal calf serum (FCS) to the DM to a final concentration of 10% (v/v). The luminal surface of each tissue section was rigorously rinsed to remove loosely-attached epithelial cells from the underlying submucosa and the resulting pooled cell suspension passed through a 70 µm cell strainer to remove tissue debris. The cell suspension was centrifuged at 300 *xg* for 5 min and resuspended in “submerged growth medium” (SGM) which comprised a 50:50 mixture of DMEM/Ham’s F-12 supplemented with 10% (v/v) FCS. The viability (typically 90–95%) of the cell suspension was assessed using the Trypan Blue exclusion assay and the cell density adjusted to 5.0 × 10^5^ cells/ml. Ten-ml of the cell suspension were seeded into T75 tissue culture flasks (5.0 × 10^6^ cells/flask) and incubated at 37 °C in a humidified atmosphere containing 5% CO_2_ and 14% O_2_ unless otherwise stated.

### Culture and differentiation of bovine bronchial epithelial cells

The BBECs were grown until 80–90% confluency (~4 days). At this point the cells were trypsinized and seeded onto 12-mm diameter, PET Thincerts of 0.4 µm pore diameter (Greiner, #665640); unless otherwise stated, membranes having a pore density of 1.0 × 10^8^ pores/cm^2^ were used. Cells were detached from the flasks using 0.25% trypsin-EDTA solution, centrifuged and resuspended in SGM to a density of 5.0 × 10^5^ cells/ml. One millilitre of SGM was added to the basolateral compartment and 0.5 ml of cell culture suspension to the apical surface (i.e. 2.5 × 10^5^ cells/insert). The epithelial cells were cultured at 37 °C in a humidified atmosphere containing 5% CO_2_ and 14% O_2_ unless otherwise stated. The following day, the basolateral and apical media were removed and replenished with fresh SGM; the apical surface was rinsed with 0.5 ml of PBS prior to the addition of fresh medium. This process was repeated every 2 to 3 days. The TEER of the cultures was measured on a daily basis using an EVOM2 epithelial voltohmmeter (World Precision Instruments, UK) according to the manufacturer’s instructions. When the TEER reached 200 Ω cm^2^ or above (~2 days), the growth medium was replaced with a 50:50 mixture of SGM and ALI medium. Air-liquid interface medium comprised a 50:50 mixture of DMEM and AEC medium (AECM; Promocell) supplemented with 10 ng/ml EGF, 100 nM RA, 6.7 ng/ml T3, 5 µg/ml insulin, 4 µl/ml bovine pituitary extract, 0.5 µg/ml hydrocortisone, 0.5 µg/ml epinephrine and 10 µg/ml transferrin (all Promocell), unless otherwise stated. When the TEER reached 500 Ω cm^2^ (indicating successful barrier formation), an ALI was generated by removing the apical medium, thereby exposing the epithelial cells to the atmosphere; this represented day 0 post-ALI. Following the formation of an ALI, the cells were fed exclusively from the basal compartment with ALI medium; apical washing, basal feeding and TEER measurements were performed every 2 to 3 days until day 21 post-ALI.

### Histology and immunohistochemistry

Cultures were fixed in 4% (w/v) paraformaldehyde for 15 min at room temperature and rinsed in PBS. The samples were dehydrated using a series of increasing ethanol concentrations, cleared with xylene, infiltrated with paraffin wax and embedded in wax blocks. Sections of 2.5 µm thickness were cut using a Thermoshandon Finesse ME + microtome and stained with H&E or PAS using standard histological techniques. For immunohistochemistry, heat-induced epitope retrieval was performed using a Menarini Access Retrieval Unit and staining conducted using a Dako Autostainer. Endogenous peroxidase was blocked with 0.3% (v/v) H_2_O_2_ in PBS. Basal cells were identified by incubation for 30 min with a 1:30 dilution of mouse anti-p63 antibody (Abcam; #ab735), application of an anti-mouse HRP-labelled polymer and visualization with a REAL EnVision Peroxidase/DAB + Detection System (Dako; #K3468). Samples were subsequently counterstained with Gill’s haematoxylin, dehydrated, cleared and mounted in synthetic resin. Tissue sections were viewed with a Leica DM2000 microscope.

### Quantification of features by light microscopy

Histological sections stained with H&E were prepared from three individual cultures derived from each of three animals. For each section, the cell layer was analysed at five randomized 40x fields of view across the strand. The thickness of the cell layer was measured at three points in each field of view using ImageJ; in addition, the number of cells forming the epithelial layer within each field of view was determined by counting the number of nuclei in each vertical section at each of the three points. The number of ciliated cells, vacuoles and pyknotic cells were also quantified within each field of view.

### Immunofluorescence microscopy

Cultures were fixed in 4% (w/v) paraformaldehyde for 15 min at room temperature and rinsed with PBS. The samples were incubated in 0.5 ml permeabilization buffer (0.5% [v/v] Triton X-100 in PBS containing 100 mg/ml sucrose, 4.8 mg/ml HEPES, 2.9 mg/ml NaCl and 600 μg/ml MgCl_2_, pH 7.2) for 10 min, washed three times with PBS and blocked for 1 h in PBST (PBS with 0.05% [v/v] Tween-20) containing 10% (v/v) normal goat serum and 1% (w/v) bovine serum albumin. The cultures were incubated with primary antibodies diluted in blocking buffer for 1 h at room temperature. The primary antibodies included mouse anti-ZO-1 antibody (Thermo Fisher; #33-9100) used at a 1:50 dilution to identify tight junction formation and rabbit anti-β-tubulin antibody (Abcam; #ab6046) used at a 1:200 dilution to identify cilia. The samples were subsequently washed three times in PBST for 2 min and incubated with secondary antibodies at a dilution of 1:400 in blocking buffer for 1 h at room temperature in the dark. The secondary antibodies included goat anti-mouse-Alexa Fluor 488 (Thermo Fisher; #A-11001) and goat anti-rabbit-Alexa Fluor 488 (Thermo Fisher; #A-11034). Mucus was visualized by incubation with a 1:200 dilution of fluorescein-labelled Jacalin for 1 h (Vector Laboratories; #FL-1151)^75^. After washing three times with PBST, nuclei were stained with 300 nM 4′,6 diamidino-2-phenylindole (DAPI) in PBS for 10 min and F-actin visualized by incubation with a 1:40 dilution of rhodamine phalloidin (Thermo Fisher; #R415) for 20 min. The samples were washed three times with PBST after each of these stains and the membranes cut from the inserts and placed onto glass slides. A drop of Vectashield mounting medium (Vector Laboratories) was added to the surface of each sample and a coverslip sealed in place using clear nail varnish. Images were acquired with a Leica DMi8 microscope for standard fluorescence microscopy. Analysis of captured images was performed using ImageJ software.

### Quantification of ciliogenesis

To quantify the degree of ciliation on the apical surface, five randomized fields of view of each β-tubulin-stained insert were acquired via a 20x objective. Images were assessed for coverage of cilia using ImageJ. A fluorescence intensity threshold was applied such that only the ciliated regions were above the threshold. The area above the threshold was measured for each image and expressed as a percentage of the total area.

### Scanning electron microscopy

Cultures were fixed in 1.5% (v/v) glutaraldehyde in 0.1 M sodium cacodylate buffer for 1 h at 4 °C. The apical and basal compartments were rinsed three times with 0.1 M sodium cacodylate buffer and the cultures post-fixed by adding 0.5 ml of 1% (w/v) osmium tetroxide to the apical surface for 1 h at room temperature. The cultures were washed three times for 10 min with distilled water, stained with 0.5% (w/v) uranyl acetate for 1 h in the dark, washed twice with distilled water and dehydrated through a series of increasing ethanol concentrations. The samples were further dehydrated in hexamethyldisilazane before being placed in a desiccator overnight. Membranes were cut from the inserts, mounted onto aluminium SEM stubs and gold sputter-coated. The cultures were analysed on a Jeol 6400 scanning electron microscope at 10 kV.

### Data Analysis

All experiments were independently performed three times using epithelial cells derived from three individual donor animals (n = 3). For quantitative analysis, three individual cultures from each donor were analysed (n = 9). Results are presented as the mean ± standard deviation. Data were statistically analysed using t-tests or Ordinary one-way ANOVAs for comparison of two groups or greater than three groups, respectively. Significance was determined by a *p*-value less than 0.05. Analyses were performed using GraphPad Prism (GraphPad Software Inc.).

## Electronic supplementary material

Supplementary information
