# Supplementary material for: Development and optimization of a differentiated airway epithelial cell model of the bovine respiratory tract
Source: Sci Rep. 2018 Jan 16;8:853. doi: 10.1038/s41598-017-19079-y (PMC5770467; doi:10.1038/s41598-017-19079-y)

Development and optimization of a differentiated airway epithelial cell model of the bovine respiratory tract

Daniel Cozens[1], Edward Grahame[1], Erin Sutherland[1], Geraldine Taylor[2], Catherine C. Berry[3] and Robert L. Davies[1*].

[1] Institute of Infection, Immunity and Inflammation, College of Medical, Veterinary and Life Sciences, University of Glasgow, Glasgow, UK

[2] The Pirbright Institute, Pirbright, Surrey, UK

[3] Institute of Molecular, Cell and Systems Biology, College of Medical, Veterinary and Life Sciences, University of Glasgow, Glasgow, UK

Corresponding Author: Robert L. Davies

Email: robert.davies@glasgow.ac.uk

Supplementary Figure Legends

Supplementary Figure S1. Histological assessment of the effect of EGF on epithelial morphology of BBEC cultures. BBEC cultures were grown for 21 days at an ALI with varying concentrations of EGF before being fixed and paraffin-embedded using standard histological techniques. Sections were cut, deparaffinised and stained as described in Fig. 1; in (**B**), red arrowheads indicate basal cells present in the suprabasal layer. Representative images are shown of BBECs grown in the presence of (i) 0, (ii) 1.0, (iii) 2.5, (iv) 5.0, (v) 10.0, (vi) 25.0 and (vii) 50.0 ng/ml EGF.

Supplementary Figure S2. Effect of EGF on cell death and vacuolation of BBEC cultures. BBEC cultures were grown for 21 days at an ALI with varying concentrations of EGF before being fixed and paraffin-embedded using standard histological techniques. Sections were cut, deparaffinised and stained using H&E. Representative images of (**A**) pyknosis and (**B**) vacuolation in epithelial cells grown in the presence of 50 ng/ml EGF are shown. Quantitative analysis (using ImageJ) of histological sections of BBEC layers grown in the presence of 0, 1.0, 2.5, 5.0, 10.0, 25.0 and 50.0 ng/ml EGF (see Fig. S1A) was performed to assess (**C**) the number of pyknotic cells and (**D**) the number of vacuoles per field of view. For each insert, the numbers of pyknotic cells and vacuoles were counted in each of five 400x fields of view evenly distributed across the sample; three inserts were analysed per growth condition and the data represents the mean +/- standard deviation from tissue derived from three different animals. Statistical significance was tested using an Ordinary one-way ANOVA: *** = $P < 0.001$.

Supplementary Figure S3. Effect of EGF on cell differentiation of BBEC cultures. BBEC cultures were grown for 21 days at an ALI with varying concentrations of EGF before fixation. The BBEC cultures were subsequently immunostained to assess (**A**) ciliation (cilia - green; F-actin - red; nuclei - blue) (**B**) mucous production (mucous - green; cilia - red; nuclei - blue) and (**C**) tight-junction formation (tight-junctions - green; nuclei - blue) or (**D**) examined by SEM. Representative images are shown of BBECs grown in the presence of (i) 0, (ii) 1.0, (iii) 2.5, (iv) 5.0, (v) 10.0, (vi) 25.0 and (vii) 50.0 ng/ml EGF.

Supplementary Figure S4. Histological assessment of the effect of RA on epithelial morphology of BBEC cultures. BBEC cultures were grown for 21 days at an ALI with varying concentrations of RA before being fixed and paraffin-embedded using standard histological techniques. Sections were cut, deparaffinised and stained using (**A**) H&E, (**B**) immunohistochemical-labelling of basal cells (p63-labelled cells display brown nuclei) and (**C**) PAS (black arrowheads indicate goblet cells). Representative images are shown of BBECs grown in the presence of (i) 0, (ii) 25, (iii) 50, (iv) 100, and (v) 250 nM RA.

Supplementary Figure S5. Effect of RA on cell differentiation of BBEC cultures. BBEC cultures were grown for 21 days at an ALI with varying concentrations of RA before fixation. The BBEC cultures were subsequently immunostained to assess (**A**) ciliation (cilia - green; F-actin - red; nuclei - blue), (**B**) mucus production (mucous - green; cilia - red; nuclei - blue) and (**C**) tight-junction formation (tight-junctions - green; nuclei - blue) or (**D**) examined by SEM. Representative images are shown of BBECs grown in the presence of (i) 0, (ii) 25, (iii) 50, (iv) 100 and (v) 250 nM RA.

Supplementary Figure S6. Histological assessment of the effect of T3 on epithelial morphology of BBEC cultures. BBEC cultures were grown for 21 days at an ALI in the absence or presence of T3 before being fixed and paraffin-embedded using standard histological techniques. Sections were cut, deparaffinised and stained as described in Fig. 1. Representative images are shown of (i) *ex vivo* bovine bronchial epithelium, and BBECs grown in the presence of (ii) 0 and (iii) 6.7 ng/ml T3. Quantitative analysis (using ImageJ) of histological sections of BBEC layers, and *ex vivo* tissue, to assess (**D**) epithelial thickness and (**E**) the number of ciliated cells per field of view, was performed as described in Fig. 1.

Supplementary Figure S7. Effect of T3 on cell differentiation of BBEC cultures. BBEC cultures were grown for 21 days at an ALI in the absence or presence of T3 before fixation. The BBEC cultures were subsequently immunostained to assess (**A**) ciliation (cilia - green; F-actin - red; nuclei - blue), (**B**) mucous production (mucous - green; cilia - red; nuclei - blue) and (**C**) tight-junction formation (tight-junctions - green; nuclei - blue) or (**D**) examined by SEM. Globules of mucus are shown on the apical surface of BBECs in (**D**). Representative images are shown of BBECs grown in the presence of (i) 0 and (ii) 6.7 ng/ml T3. Quantitative analysis (using ImageJ) of ciliation of the apical surface of BBEC cultures was performed (**E**) using fluorescence intensity thresholding of immunostained cultures and (**F**) by counting the number of ciliated cells per field of view in H&E-stained sections as described in Fig. 2.

Supplementary Figure S8. Histological assessment of the effect of oxygen tension on epithelial morphology of BBEC cultures. BBEC cultures were grown for 21 days at an ALI in the presence of varying oxygen tensions before being fixed and paraffin-embedded using

standard histological techniques; samples of *ex vivo* tissue were also taken from the donor animal. Sections were cut, deparaffinised and stained as described in Fig. 1. Representative images are shown of (i) *ex vivo* bovine bronchial epithelium, and BBECs grown in the presence of (ii) 7%, (iii) 14% and (iii) 21% oxygen tension. Quantitative analysis (using ImageJ) of histological sections of BBEC layers, and *ex vivo* tissue, to assess (**D**) epithelial thickness and (**E**) the number of cell layers comprising the epithelium, was performed as described in Fig. 1.

Supplementary Figure S9. Effect of oxygen tension on cell differentiation of BBEC cultures. BBEC cultures were grown for 21 days at an ALI in the presence of varying oxygen tensions before fixation. The BBEC cultures were subsequently immunostained to assess (**A**) ciliation (cilia - green; F-actin - red; nuclei - blue), (**B**) mucous production (mucous - green; cilia - red; nuclei - blue) and (**C**) tight-junction formation (tight-junctions - green; nuclei - blue) or (**D**) examined by SEM. Representative images are shown of BBECs grown in the presence of (i) 7%, (ii) 14% and (iii) 21% oxygen tension. Quantitative analysis (using ImageJ) of ciliation of the apical surface of BBEC cultures was performed (**E**) using fluorescence intensity thresholding of immunostained cultures and (**F**) by counting the number of ciliated cells per field of view in H&E-stained sections as described in Fig. 2. Statistical significance was tested using an Ordinary one-way ANOVA: * = $P < 0.05$; ** = $P < 0.01$.

Supplementary Figure S1

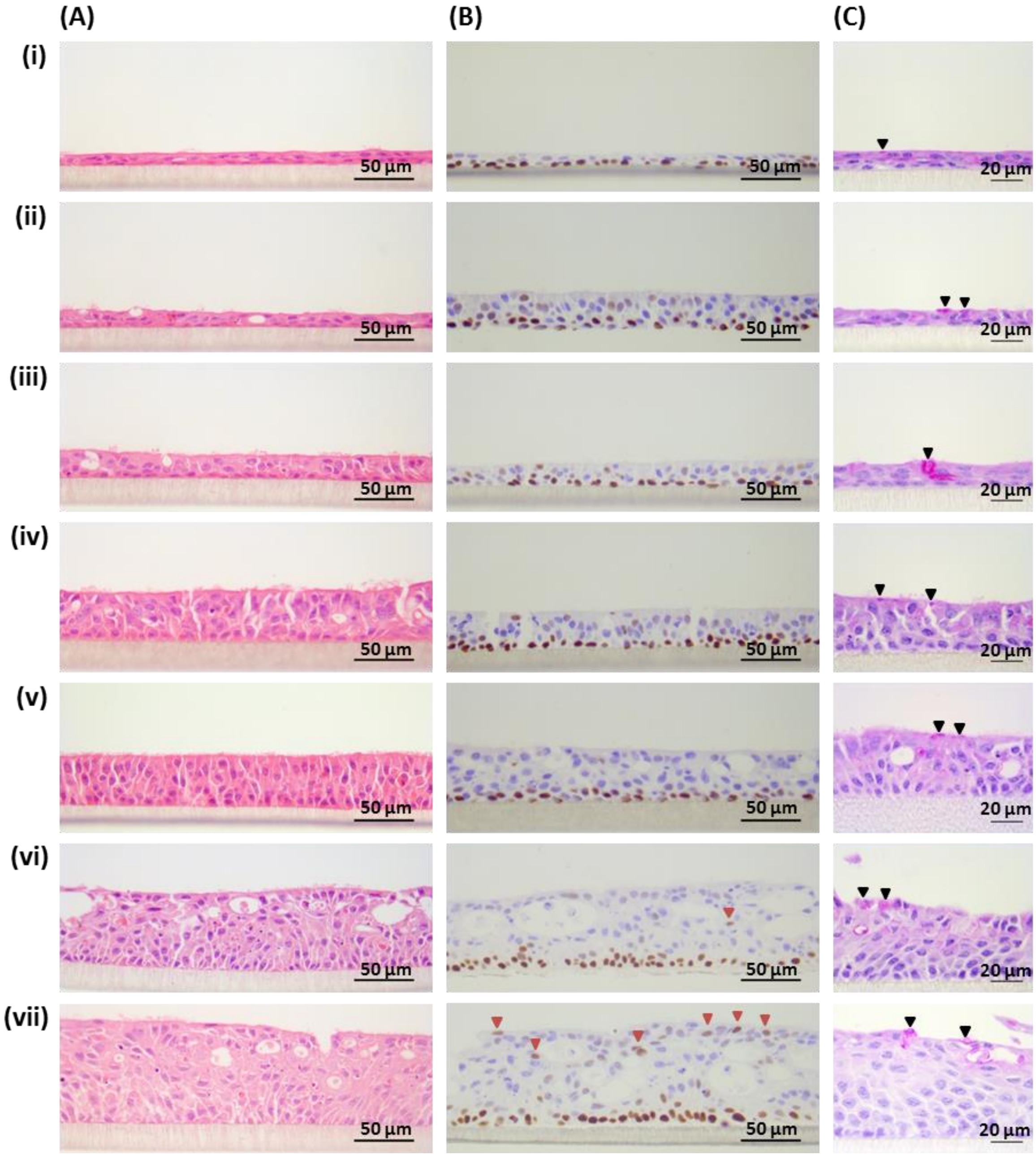

Supplementary Figure S2

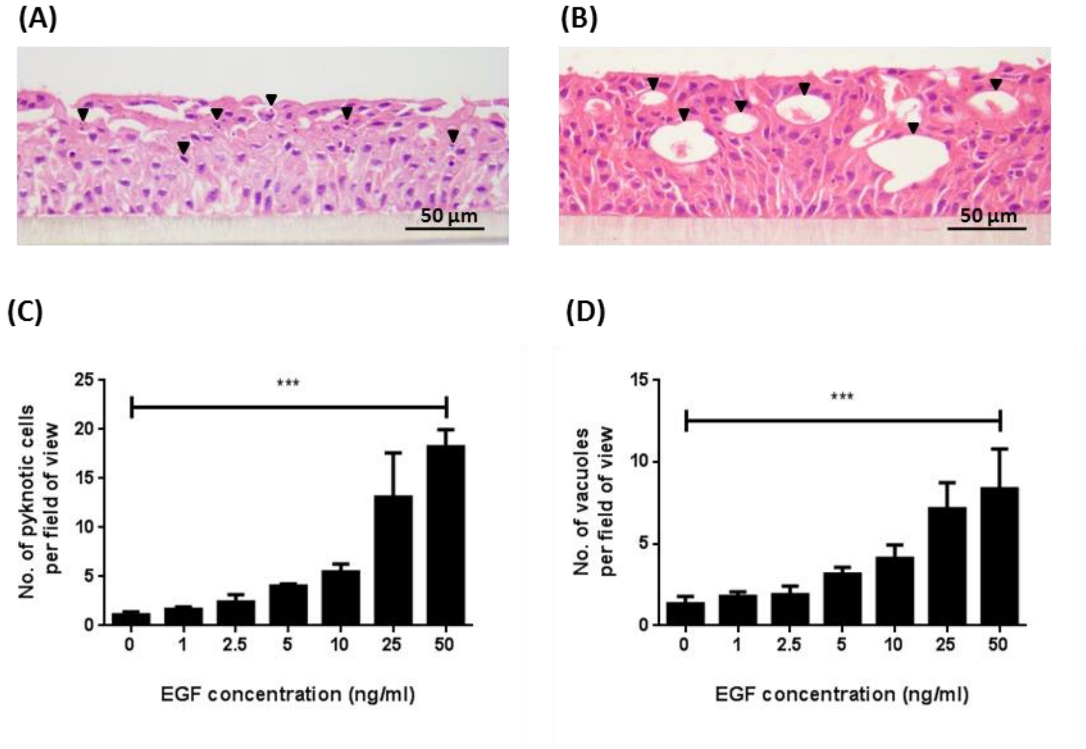

Supplementary Figure S3

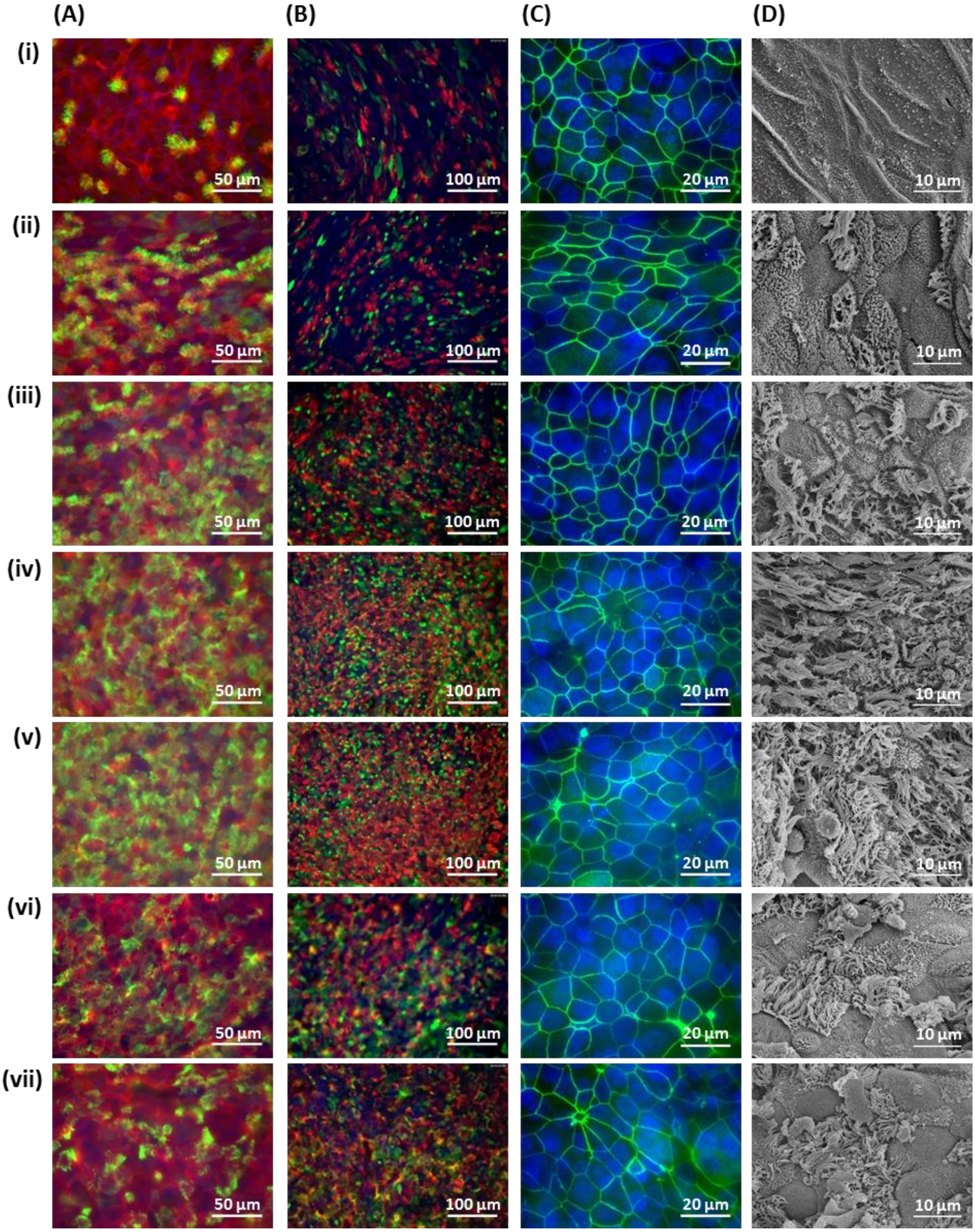

Supplementary Figure S4

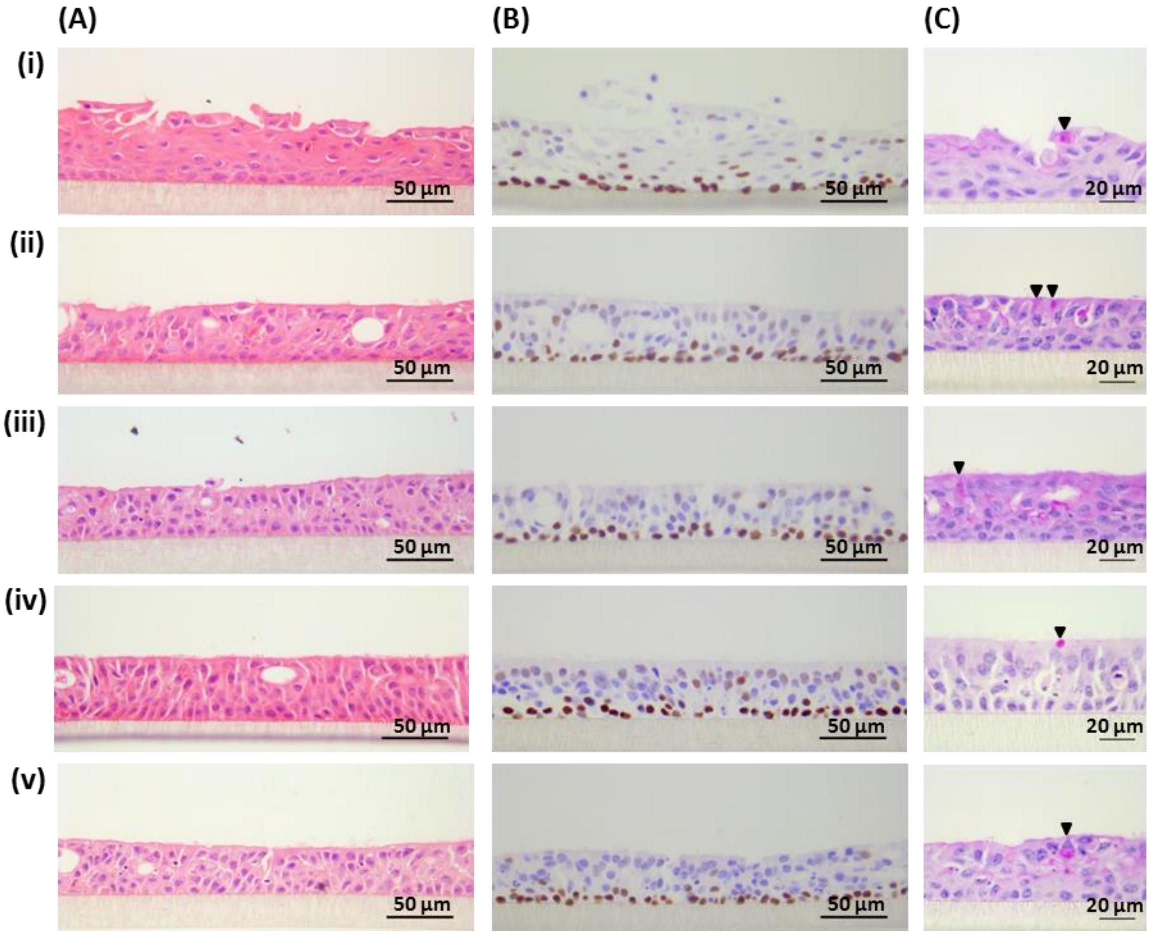

Supplementary Figure S5

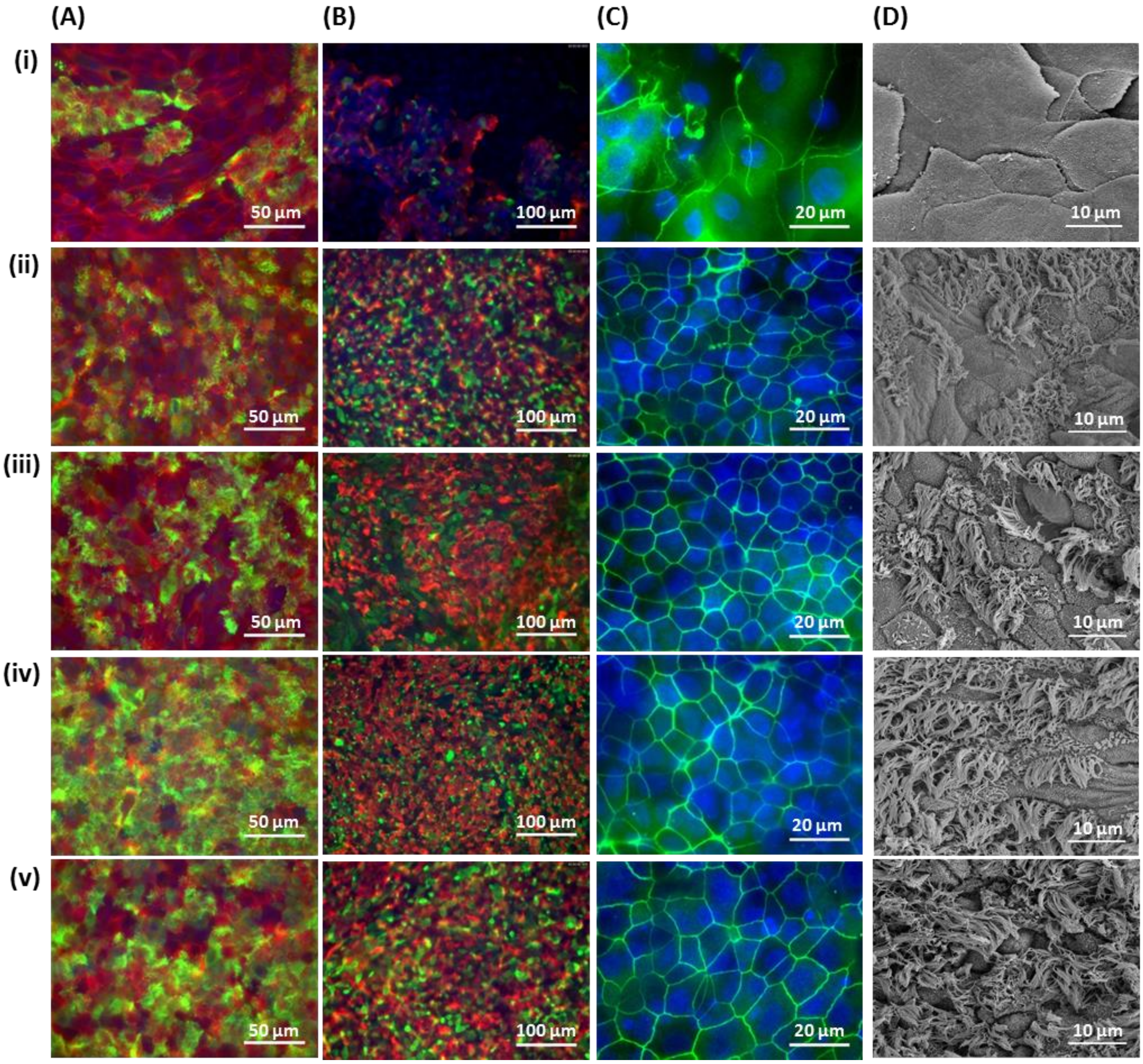

Supplementary Figure S6

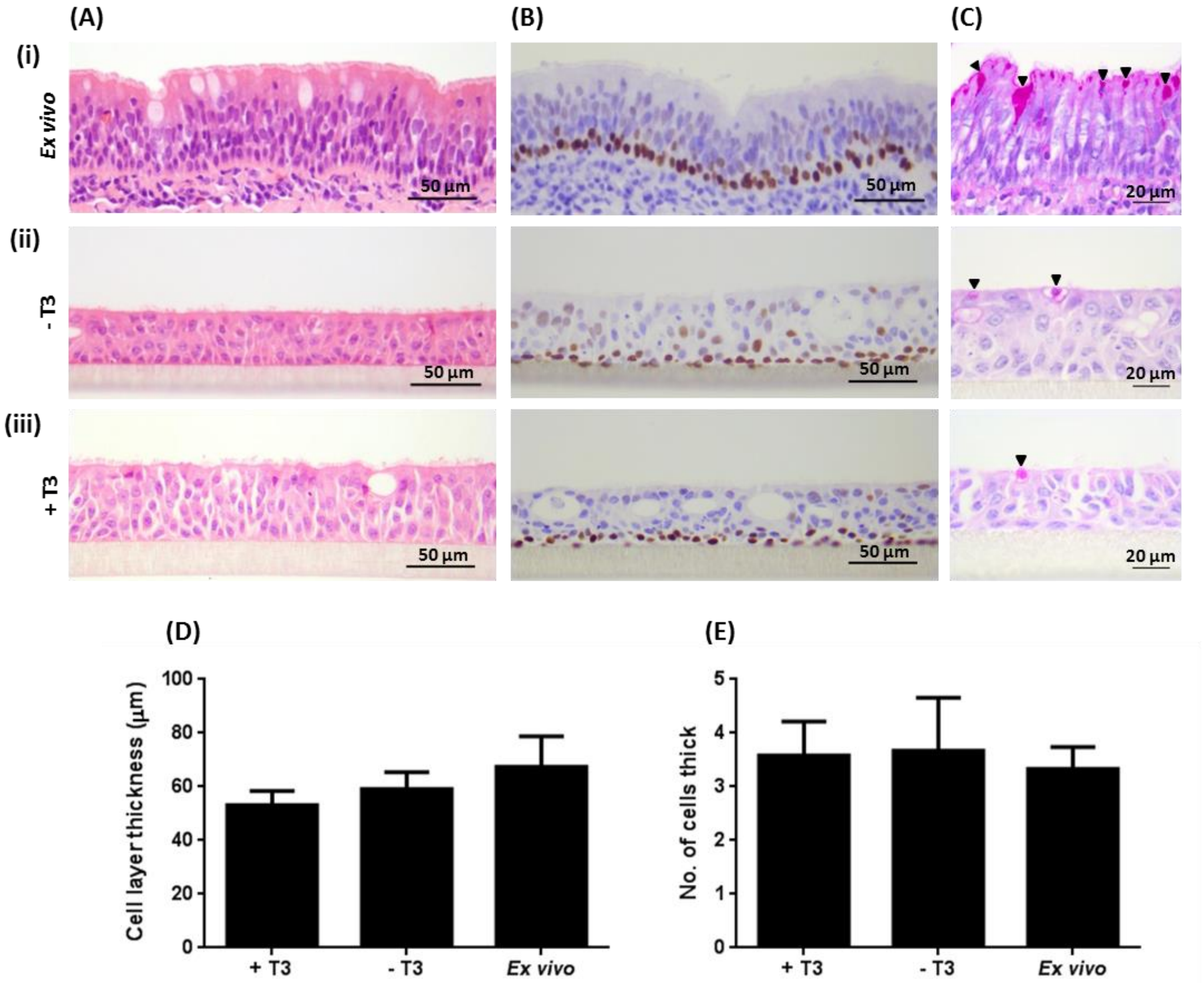

Supplementary Figure S7

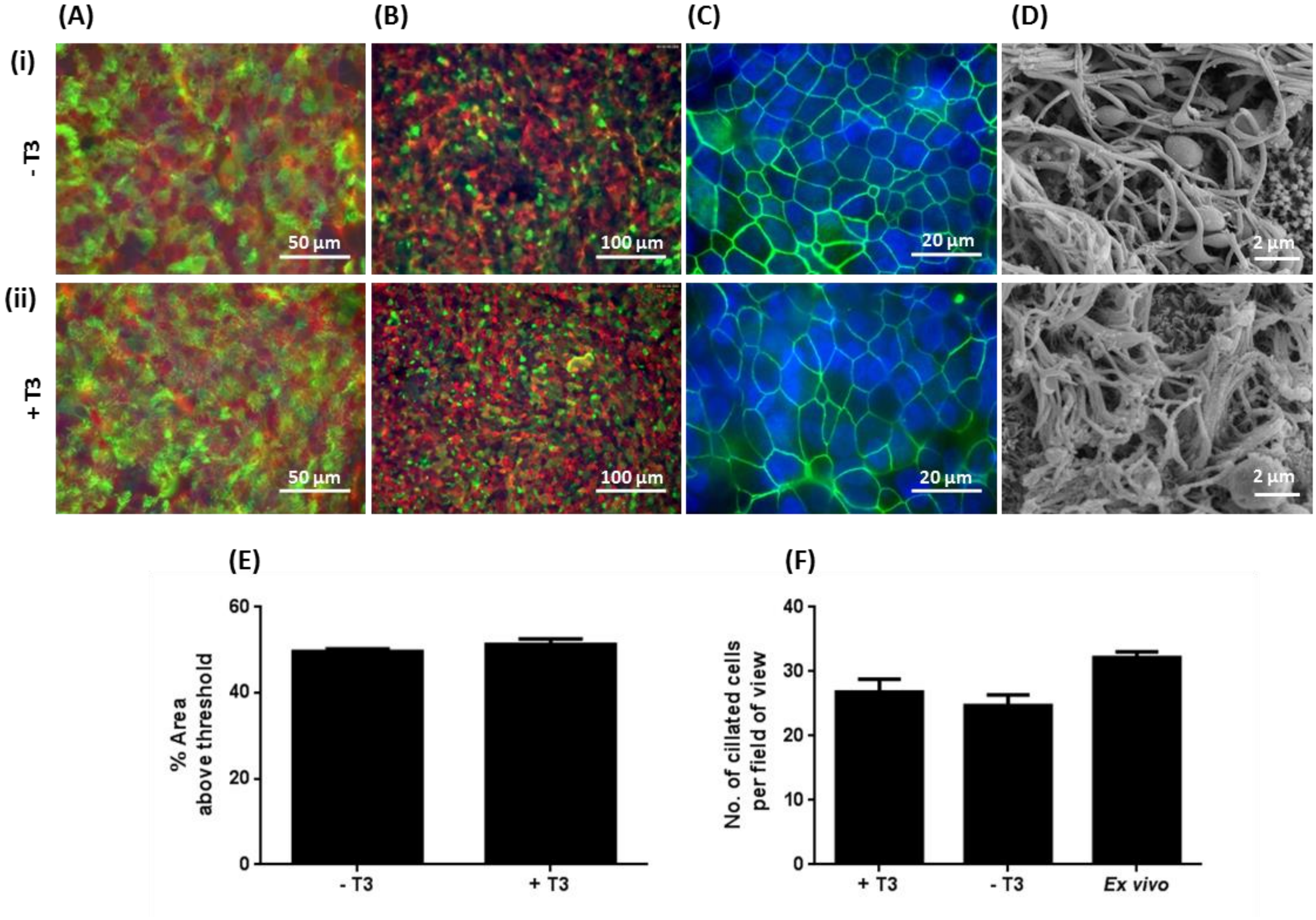

Supplementary Figure S8

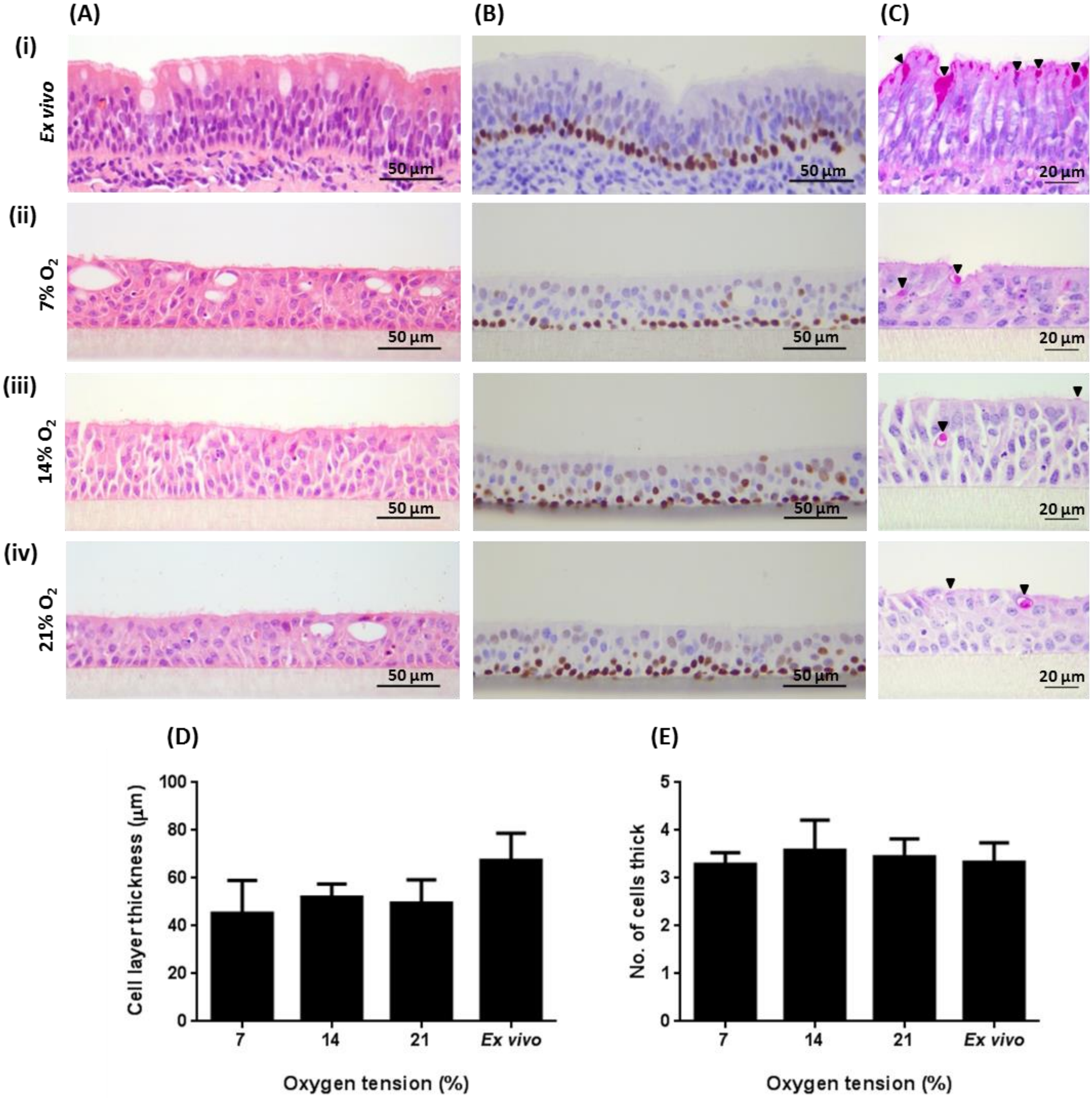

Supplementary Figure S9

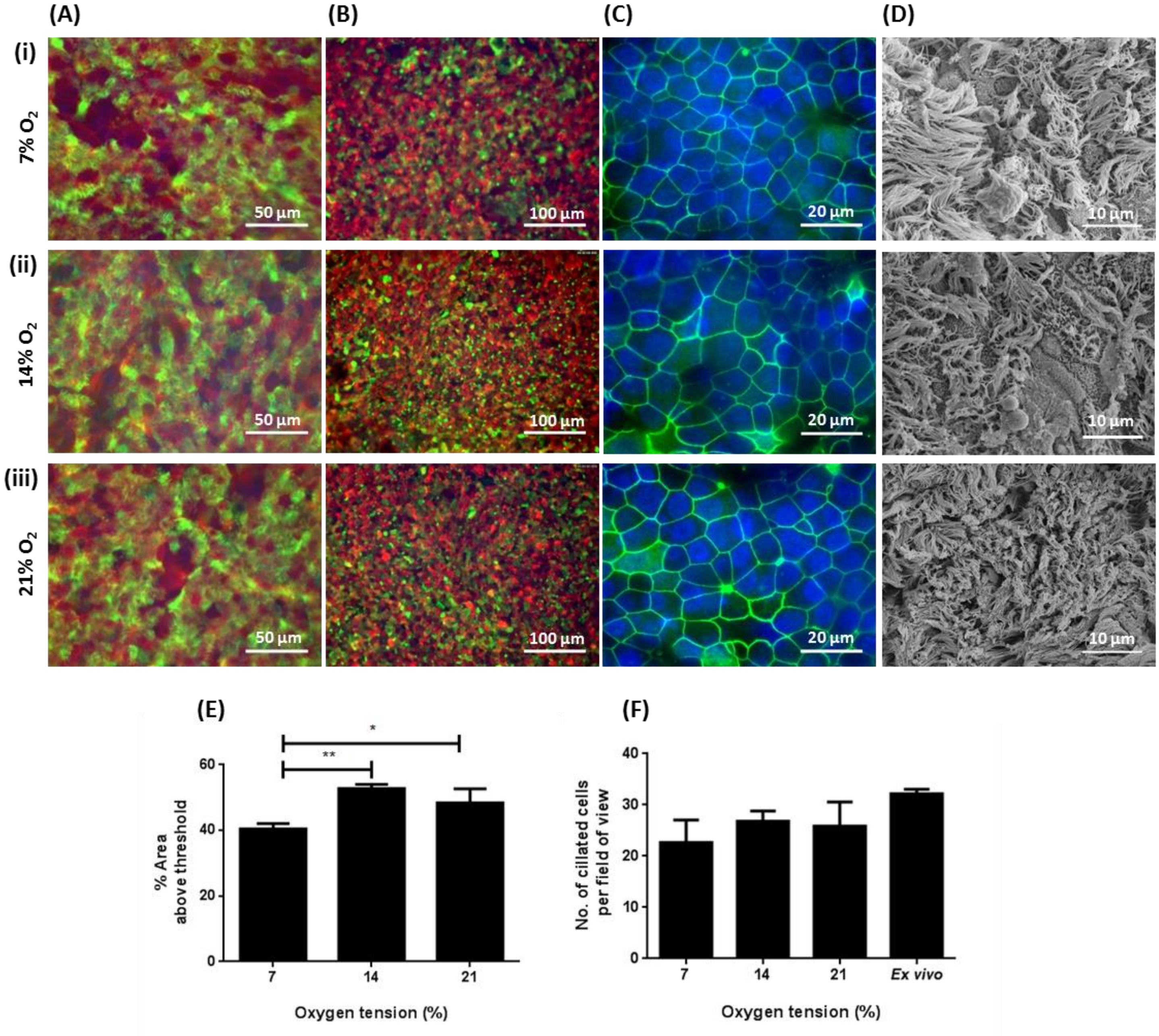

Supplement: Supplementary file 1 — Supplementary information [file 41598_2017_19079_MOESM1_ESM.pdf]
